# Regulator of G-Protein Signaling 14 (RGS14) Is a Selective H-Ras Effector

**DOI:** 10.1371/journal.pone.0004884

**Published:** 2009-03-25

**Authors:** Francis S. Willard, Melinda D. Willard, Adam J. Kimple, Meera Soundararajan, Emily A. Oestreich, Xiaoyan Li, Nathaniel A. Sowa, Randall J. Kimple, Declan A. Doyle, Channing J. Der, Mark J. Zylka, William D. Snider, David P. Siderovski

**Affiliations:** 1 Department of Pharmacology, University of North Carolina, Chapel Hill, North Carolina, United States of America; 2 UNC Neuroscience Center, University of North Carolina, Chapel Hill, North Carolina, United States of America; 3 Lineberger Comprehensive Cancer Center, University of North Carolina, Chapel Hill, North Carolina, United States of America; 4 Structural Genomics Consortium, Oxford University, Oxford, United Kingdom; Universidade Federal do Rio de Janeiro (UFRJ), Instituto de Biofísica da UFRJ, Brazil

## Abstract

**Background:**

Regulator of G-protein signaling (RGS) proteins have been well-described as accelerators of Gα-mediated GTP hydrolysis (“GTPase-accelerating proteins” or GAPs). However, RGS proteins with complex domain architectures are now known to regulate much more than Gα GTPase activity. RGS14 contains tandem Ras-binding domains that have been reported to bind to Rap- but not Ras GTPases *in vitro*, leading to the suggestion that RGS14 is a Rap-specific effector. However, more recent data from mammals and *Drosophila* imply that, *in vivo*, RGS14 may instead be an effector of Ras.

**Methodology/Principal Findings:**

Full-length and truncated forms of purified RGS14 protein were found to bind indiscriminately *in vitro* to both Rap- and Ras-family GTPases, consistent with prior literature reports. In stark contrast, however, we found that in a cellular context RGS14 selectively binds to activated H-Ras and not to Rap isoforms. Co-transfection / co-immunoprecipitation experiments demonstrated the ability of full-length RGS14 to assemble a multiprotein complex with components of the ERK MAPK pathway in a manner dependent on activated H-Ras. Small interfering RNA-mediated knockdown of RGS14 inhibited both nerve growth factor- and basic fibrobast growth factor-mediated neuronal differentiation of PC12 cells, a process which is known to be dependent on Ras-ERK signaling.

**Conclusions/Significance:**

In cells, RGS14 facilitates the formation of a selective Ras·GTP-Raf-MEK-ERK multiprotein complex to promote sustained ERK activation and regulate H-Ras-dependent neuritogenesis. This cellular function for RGS14 is similar but distinct from that recently described for its closely-related paralogue, RGS12, which shares the tandem Ras-binding domain architecture with RGS14.

## Introduction

Many extracellular signaling molecules exert their cellular effects through activation of G protein-coupled receptors (GPCRs) [1]–[3]. GPCRs are seven transmembrane spanning proteins coupled to a membrane-associated heterotrimeric complex that is comprised of a GTP-hydrolyzing Gα subunit and a Gβγ dimeric partner [1], [2]. Agonist-bound GPCRs catalyze the release of GDP, and subsequent binding of GTP, by the Gα subunit [1], [2]. On binding GTP, conformational changes within the three ‘switch’ regions of Gα facilitate the release of the Gβγ dimer. Gα·GTP and Gβγ subunits regulate the activity of target effector proteins such as adenylyl cyclases, phospholipase C isoforms, ion channels, and phosphodiesterases, which in turn regulate multiple downstream signaling cascades that initiate key biological processes such as development, vision, olfaction, cardiac contractility, and neurotransmission [1]–[3]. The intrinsic GTP hydrolysis (GTPase) activity of Gα resets the cycle by forming Gα·GDP – a nucleotide state with low affinity for effectors but high affinity for Gβγ. Reassociation of Gα·GDP with Gβγ reforms the inactive, GDP-bound heterotrimer which completes the cycle [1], [2]. Thus, the duration of G-protein signaling through effectors is thought to be controlled by the lifetime of the Gα subunit in its GTP-bound form [2], [4]. The lifetime of Gα·GTP is modulated by RGS (regulators of G-protein signaling) domain-containing proteins [4]. The RGS domain is a ∼120 amino-acid nine-alpha helical bundle [5], [6] that contacts Gα subunits and thereby dramatically accelerates GTPase activity [7], [8]. Many RGS proteins catalyze rapid GTP hydrolysis by isolated Gα subunits *in vitro* and attenuate or modulate GPCR-initiated signaling *in vivo* [4], [5], [8]; accordingly, RGS proteins are considered key desensitizers of heterotrimeric G-protein signaling pathways [4], [8].

It has become apparent that the signature RGS domain is a modular protein fold found in multiple biological contexts [4], [8]. The identification of multidomain RGS proteins has led to a new appreciation of these molecules as being more than just GAPs for Gα subunits [4], [8], [9]. RGS14 is an RGS protein with multiple signaling regulatory elements, as it contains an RGS domain, tandem RBDs (Ras-binding domains), and a GoLoco motif [10], [11]. In addition to the RGS domain of RGS14 acting as a GAP for Gα_i/o_ subunits [11]–[13], the GoLoco motif of RGS14 functions as a guanine nucleotide dissociation inhibitor (GDI) for Gα_i1/i3_ subunits [14], [15]. Beyond regulation of heterotrimeric Gα signaling, RGS14 is also reported to bind to activated monomeric G-proteins. An early yeast two-hybrid analysis of interactions between RGS14 and Ras-family GTPases reported a selective interaction between RGS14 and activated Rap1B, but not H-Ras [11]; *in vitro* experiments have also shown RGS14 binding in a nucleotide-dependent manner to the small GTPases Rap1 and Rap2 but not Ras [11], [16]–[18]. Based on these results, it has been suggested that RGS14 may be a direct effector of Rap *in vivo*. However, subsequent to this initial identification of Rap (and not Ras) as a small GTPase binding target of RGS14, additional studies have suggested that Ras may also bind to RGS14. Kiel *et al.* [16] found that RGS14 binds preferentially to both activated Rap1B and activated H-Ras *in vitro*, and that this interaction is mediated by the first RBD of RGS14. Similarly, Formstecher *et al.* [19] identified Loco (the *Drosophila* RGS12/14 orthologue) in a screen for binding partners of activated Rap1, Rap2, and Ras1. Finally, we have recently discovered that RGS12, the mammalian paralogue of RGS14, binds specifically to activated H-Ras in cells [20]. Collectively, these results suggest that RGS14 may bind to Rap and/or Ras GTPases. In addition to binding activated H-Ras, we found that RGS12 promotes a differentiated phenotype in both PC12 cells and embryonic DRG neurons by organizing a Ras, Raf, MEK, and ERK signal transduction complex [20]. The requirement for RGS12 in nerve growth factor (NGF)-induced neuritogenesis of PC12 cells and axonal growth of embryonic DRG neurons suggests that the related protein RGS14 may play a similar role in coordinating Ras-dependent signals that are required for promoting and/or maintaining cellular differentiation [20].

Our aim with these present studies was to resolve the discordant ideas regarding the monomeric G-protein selectivity of RGS14, as well as to establish a cellular role for such RGS14/monomeric G-protein interaction(s). Here, we demonstrate that full-length and truncated forms of RGS14 bind promiscuously to Rap and Ras GTPases *in vitro*, consistent with earlier reports. In cells, however, RGS14 selectively binds to activated H-Ras and not Rap nor most other Ras family isoforms. Additionally, RGS14 facilitates the formation of a Raf/MEK/ERK multiprotein complex that is dependent on activated H-Ras. Furthermore, small interfering RNA (siRNA)-mediated downregulation of RGS14 inhibits both NGF- and basic fibrobast growth factor (bFGF)-mediated neuritogenesis of PC12 cells, both processes known to require Ras-ERK signaling. These results suggest that RGS14 may regulate neuronal differentiation by the selective organization of a Ras·GTP-dependent Raf, MEK, and ERK signal transduction complex *in vivo*.

## Materials and Methods

### Materials

2.5S mouse NGF was from Roche (Indianapolis, IN). Human basic FGF was from Sigma (St. Louis, MO). Antibodies: Anti-β-actin (AC-74) and anti-FLAG M2 (Sigma), anti-ERK1/2 and anti-phospho-ERK1/2(T202, Y204) (Cell Signaling Technologies; Danvers, MA), anti-HA-HRP 3F10 and anti-myc 9E10 (Roche), anti-myc-HRP 4A6 (Millipore, Billerica, MA), anti-rabbit IgG HRP and anti-mouse IgG HRP (GE Healthcare; Piscataway, NJ), and anti-Rap2 (BD Biosciences, San Jose, CA). All siRNAs were from Dharmacon (Lafayette, CO). siRNA sequences: rat RGS14 siGENOME SMARTpool (sense strand sequences: duplex 1, 5′-GUACGGAUCUCUGCUAAGC-3′; duplex 2, 5′-GAAAGUCACUGCCGCUCGG-3′; duplex 3, 5-GGGAAGUACUGCUGCGUGU-3′; duplex 4, 5′-CCGCAAGUCCUUUCGUAGA-3′). Rat RGS12(duplex 2) siRNA and the control ‘non-specific’ siRNA are described in [20]. Unless elsewhere specified, all additional reagents were of the highest quality obtainable from Sigma or Fisher (Pittsburgh, PA).

### Molecular biology

All DNA constructs were created using standard methods or obtained for these studies as described in Table S1. Site-directed mutagenesis was performed using the QuikChange system (Stratagene, La Jolla, CA). All DNA constructs were verified by DNA sequencing (Agencourt, Beverly, MA).

### Cell culture and transfection

HEK293T and PC12 cells were cultured and transfected as described previously [20]. In PC12 experiments, pBabe-puro retrovirus expression vectors encoding constitutively-actived B-Raf(V600E) and H-Ras(G12V) were co-transfected with siRNA using LipofectAMINE 2000 (Invitrogen, Carlsbad, CA), essentially as described [20]. For co-transfections, 300 ng DNA and 150 pmol siRNA were used in a final volume of 1 ml, in 12-well plates. Neurite length was quantified after 3 days (B-Raf) or 4 days (H-Ras). We were unable to obtain an antibody capable of specifically detecting endogenous levels of murine RGS14, and thus unable to directly test for RGS14 protein knockdown in PC12 cells. To obviate this problem, we initially tested the specificity and efficacy of siRNA duplexes using HEK293T cells. HEK293T cells were plated in antibiotic-free DMEM at 165,000 cells per well in a total volume of 1 ml per well of a 12-well plate. The following day, cells were transfected with epitope-tagged RGS14 expression constructs: 20 ng myc-tagged rat RGS14 were transfected using FuGENE-6 (Roche) as described [20], [21]. pcDNA3.1 was used to balance DNA amounts to a total of 1.5 µg per well. Five hours after transfection, medium was removed and cells were equilibrated in 1 ml OPTI-MEM-I (Invitrogen) for one hour. Subsequently, cells were transfected with siRNA duplexes using LipofectAMINE 2000, as described [20]. Five hours after siRNA transfection, the medium was changed to 2 ml of antibiotic-free medium per well.

### Quantitative PCR

For qRT-PCR experiments that validated the specificity and efficacy of RNAi-mediated knockdown, PC12 were transfected with siRNA duplexes as described above. 48 hours post transfection, cells were washed once with PBS and then scraped and resuspended in 500 µl of PBS. Total RNA extraction and subsequent RT-PCR was performed exactly as previously described [22] using gene-specific primers and 6-carboxyfluorescein (FAM) and 6-carboxytetramethylrhodamine (TAMRA) dual labeled probes. Primer sequences: Rat β-actin: forward, 5′-TGCCTGACGGTCAGGTCA-3′; reverse, 5′-CAGGAAGGAAGGCTGGAAG-3′; probe 5′-FAM-CACTATCGGCAATGAGCGGTTCCG-TAMRA-3′; Rat RGS12: forward, 5′-CATGTC CCTGCACATGACAA-3′; reverse, 5′-TGGCTT TGCTGCACAGGAAT-3′; probe, 5′-FAM-AAAATCTCCCGGGCCCTGTAAGAG-TAMRA-3′; Rat RGS14: forward, 5′-CTACCT GACATTAAGGTCTACC; reverse, 5′-ACGGTG CAGTCCTGATCCA-3′; probe, 5′-FAM-CAG GGCCTTCTGTTCTTTGCCCA-TAMRA-3′. The number of cycles until threshold (C_t_) was determined using an ABI Prism 7700 Sequence Detector System (Applied Biosystems, Foster City, CA). To normalize for variation in the total number of cells and the efficiency of the mRNA extraction, the C_t_ value for β-actin *C_t_(β-actin)* was subtracted from C_t_ values for RGS12 *C_t_(RGS12)* and RGS14 *C_t_(RGS14)*. The change in RGS12 and RGS14 expression was then determined relative to cells treated with non-specific siRNA using the 2^−(ΔΔCt)^ method [23]. For example, RGS14 expression levels relative to control-treated (non-specific siRNA) samples were determined using the following equation:
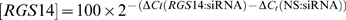
where

Δ*C_t_*(*RGS14*:siRNA) = *C_t_*(*RGS14*)−*C_t_*(*β-actin*) and
Δ*C_t_*(NS:siRNA) = *C_t_*(NS)−*C_t_*(*β-actin*). All experiments were performed in triplicate.

### Neurite outgrowth

PC12 neurite outgrowth was quantified essentially as described [20]. For co-transfection experiments involving siRNA knockdown along with activated H-Ras/B-Raf expression, percentages of cells containing neurites longer than one cell body were also determined. Bright-field photomicrographs of PC12 cells were obtained as described [20]. To enhance the visibility of neurites, micrographs were processed for publication using Adobe Photoshop (v7.0.1); the following commands were used sequentially: greyscale, autocontrast, autolevel, curves (50% input, 25% output).

### Bimolecular fluorescence complementation

HEK 293T cells were seeded at 200,000 cells per well in 6-well dish. Cells were transfected with a total of 1 µg of DNA using FuGENE-6 (3 µl/µg of DNA). Empty pcDNA3.1 vector DNA was used to maintain a constant amount of total DNA per well. Forty-eight hours post-transfection, epifluorescence images were acquired using an Olympus I×70 fluorescence microscope with a Q-Fire CCD camera (Olympus, USA). All digital images were acquired using 14.1 sec exposures at 20× magnification and imported into Photoshop. Digital images were saved as “portable network graphics” (PNG) files and imported into MATLAB 2007a (The MathWorks, Inc. Natick, MA). Pixels with greater than 40 units of intensity in the green channel were considered to be fluorescent. The percent of fluorescent pixels for each experiment was then quantified. All experiments were repeated three times. Control experiments were performed to demonstrate the specificity of fluorescence complementation: *e.g.*, YFP_N_ alone was unable to complement YFP_C_-RGS14 and YFP_C_ alone was unable to complement YFP_N_-H-Ras(G12S).

### Western blotting

Protein/cell lysate electrophoresis and immunoblotting was performed as described [20]. Images were scanned using a Perfection 1200/GT-7600 scanner (Epson; Long Beach, CA). Quantification of immunoblots was performed using the Scion Image measure function (Scion Corp, Frederick, MD).

### Immunoprecipitation

Immunoprecipitation experiments were conducted essentially as described [20], with the minor modification that all lysis and wash buffers contained 20 mM MgCl_2_. Immunoprecipitations were carried out by incubation of cell lysates with antibodies overnight at 4°C, and immune complex precipitation was achieved by incubation with 40 µl of protein A/G agarose (Santa Cruz) for one hour before washing and elution. All washing and elution steps were performed chromatographically using micro Bio-Spin columns (BioRad, Hercules, CA), as described [24]. For some experiments, pre-clearing of lysates was used to reduce non-specific binding. Pre-clearing was performed by incubating lysates at 4°C with 50 µl protein A/G agarose for 2 h. Agarose beads were removed from lysates using micro Bio-Spin columns.

### GST co-precipitations

Glutathione agarose was prepared by resuspension of dry beads in excess lysis buffer (20 mM TRIS/HCl pH 7.5, 100 mM NaCl, 20 mM MgCl_2_, 1 mM EGTA, 1% (v/v) Triton X-100, and Complete Mini protease inhibitors (Roche)). Beads were swollen for 10 min and then washed three times by brief centrifugation, and prepared for use as a 50% (v/v) slurry. HEK293T cells were transfected with expression plasmids for small GTPases (1500 ng DNA/well of a 6 well dish) as described [20], [21]. Cells were lysed in 750 µl per well of lysis buffer; generally one well per experimental condition was sufficient. Lysates were prepared as described [20], and then pre-cleared for 2–4 h at 4°C with 100 µl per sample of glutathione agarose beads. Beads were removed from lysates using micro Bio-Spin columns. 500 pmol GST-fusion protein was added per lysate sample, and aliquots of this mixture were taken as ‘Loading Control’ samples for SDS-PAGE. Lysate/GST-fusion protein mixtures were then incubated overnight at 4°C, with gentle agitation. Subsequently, GST-fusion proteins and bound GTPases were precipitated with 40 µl of glutathione agarose by incubation at 4°C, for 1 h, with gentle agitation. Beads were applied to micro Bio-Spin columns and washed by gravity flow with 4×1 ml lysis buffer, followed by a final brief centrifugation (16,300×g, 30 s). Protein was eluted with 60 µl Laemmli buffer and centrifugation (16,300×g, 30 s).

### Protein purification

The bacterial expression vectors pNIC-SGC(RGS14(RBD1.RBD2)) or pPROEXHTb(H-Ras) were separately expressed in BL21(DE3) *E. coli*, essentially as described [25]. One liter cultures of terrific broth were grown at 37°C until an OD_600 nm_ of 1 was reached. Protein was induced with 0.5 mM isopropyl β-D-thiogalactoside for 12 h at 22°C. Cells were harvested by centrifugation at 9000×g for 20 min and resuspended in lysis buffer (50 mM HEPES, pH 7.5, 300 mM NaCl, 5% (v/v) glycerol and 10 mM imidazole) and frozen at −80°C until further use. Cell pellets containing H-Ras were resuspended in the above buffer supplemented with 2 mM MgCl_2_. Frozen cell pellets were thawed in the presence of one EDTA-free Complete™ protease inhibitor tablet per liter (Roche) and then were lysed using an Emulsiflex C5 high pressure homogenizer (Avestin; Ottawa, Canada). Poly(ethyleneimine) was then added to a final concentration of 0.15% (v/v) and insoluble debris was removed by centrifugation for 45 min at 15000 rpm using a JA-17 rotor (Beckman Coulter, Fullerton, CA). Protein was extracted from clarified supernatant by affinity-tag purification using Ni-NTA (Ni^2+^-nitrilotriacetate) resin (Qiagen, Valencia, CA). H-Ras purification buffers were supplemented with 2 mM MgCl_2_. Supernatant was passed over Ni-NTA resin, which was then washed with 30 column volumes of lysis buffer and 5 column volumes of wash buffer (50 mM HEPES, pH 7.5, 300 mM NaCl, 5% (v/v) glycerol and 25 mM imidazole). Protein was eluted from the resin with 5 column volumes of elution buffer (50 mM HEPES, pH 7.5, 300 mM NaCl, 5% (v/v) glycerol and 250 mM imidazole). Eluted protein was purified further by gel filtration chromatography using a Sephadex S200 16/60 column (GE Healthcare). RGS14(RBD1.RBD2) was subject to gel filtration using 50 mM HEPES, pH 7.5, 300 mM NaCl, and 0.5 mM Tris(2-carboxyethyl)phosphine hydrochloride. H-Ras was treated for 12 h with 50 U of calf intestinal phosphatase, 10 mM EDTA, TEV protease and 1 mM GPPNHP at 4°C and then subjected to gel filtration using 50 mM HEPES pH 7.5, 150 mM NaCl, 2 mM MgCl_2_, 0.5 mM TCEP. Proteins were concentrated using 10 kDa cut-off Amicon ultra filters (Millipore, Burlington, MA). GST-RGS14-His_6_ was purified as described [26]; all other GST-fusion proteins were purified as described [24].

### Isothermal titration calorimetry

Isothermal titration calorimetry (ITC) measurements were carried out at 20°C using a VP-ITC MicroCalorimeter (MicroCal; Northampton, MA). Guanine nucleotide-loaded H-Ras and RGS14(RBD1.RBD2) were each in a solution of 20 mM HEPES, pH 7.5, 150 mM NaCl, 1 mM MgCl_2_ and 0.5 mM TCEP, which was degassed in a ThermoVac apparatus (MicroCal). ITC experiments were performed by stepwise titration of RGS14(RBD1.RBD2) (300 µM) into an adiabatic cell containing H-Ras (20 µM), and the heat energy change accompanying the reaction was detected upon each injection by comparison with a reference cell. Protein solution was placed in the 1.4 ml calorimeter cell and stirred to ensure rapid mixing, and 10 µl aliquots of the titrant were injected over 10 s with a 4 min interval between each injection until saturation. The titrant injected into buffer alone was used as a negative control. Heat change data was determined by subtracting values obtained when RGS14 was titrated into buffer alone. Subsequently, data was integrated and plotted against the molar ratio of H-Ras/RGS14 and analyzed as a non-linear least-squares fit. Data were analyzed using a single binding site model with the ORIGIN software package supplied by MicroCal.

### Yeast two-hybrid assay

Yeast two-hybrid assays were performed essentially as described [27] using PJ69-4A *S. cerevisiae* [28]. “Bait” constructs in pGBT9 encoded the H-Ras and Rap1B GTPases fused to the Gal4p DNA binding domain, as described [29], [30]. “Prey” constructs in pACTII [31] encoded the Gal4p activation domain fused to the isolated RBDs of human Raf-1 or rat RGS14.

### Statistics

Graphical and statistical analysis was performed using Prism 4.0 (GraphPad, San Diego, CA). All data presented are representative of three or more independent experiments.

## Results

### RGS14 binds promiscuously *in vitro* to Ras and Rap isoforms

**Figure 1:**
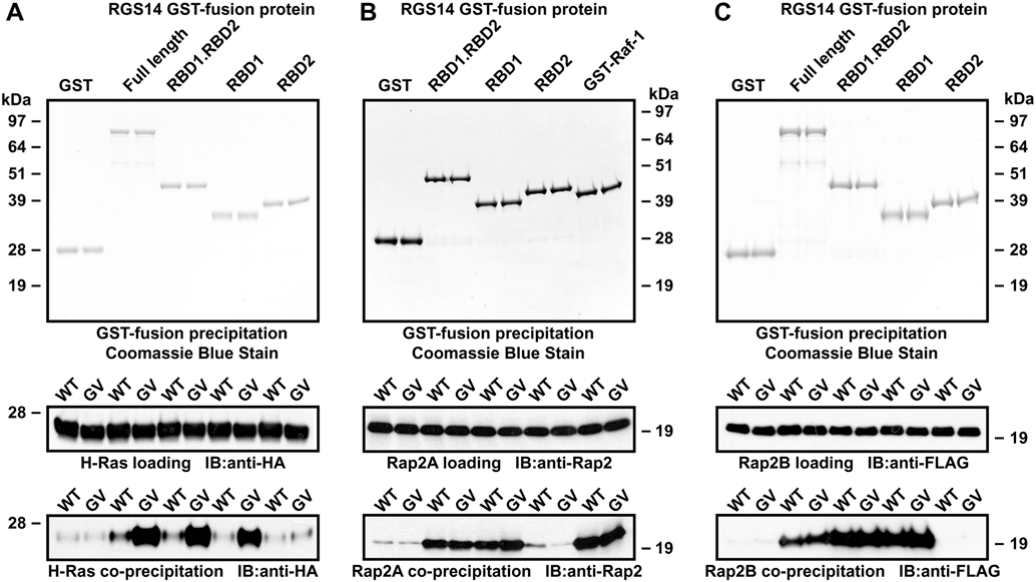
The first Ras-binding domain of RGS14 interacts with Ras and Rap family GTPases *in vitro*. GST-fusion proteins of indicated full-length or truncated RGS14 were incubated with lysates from HEK293T cells transfected with wild-type (WT) or mutationally-activated (GV) HA-tagged H-Ras (A), Rap2A (B), or FLAG-tagged Rap2B (C). Protein complexes were precipitated with glutathione agarose, washed, and resolved by SDS-PAGE and immunoblot (IB) (bottom panels). Experimental samples were also analyzed by immunoblot to ensure equivalent loading of GTPases (middle panels). Precipitation of GST-fusion proteins was confirmed by SDS-PAGE and Coomassie Blue staining (top panels). Data are representative of 3 or more independent experiments.

RGS14 contains two putative RBDs in tandem, and has previously been demonstrated to interact preferentially with the GTP-bound forms of Rap1 and Rap2 but not Ras [11], [17], [18]. However, one group has used ITC to show that the isolated tandem RBDs of RGS14 have micromolar binding affinities for both recombinant H-Ras and Rap1B [16]. To determine the selectivity of RGS14 for Ras-family GTPases *in vitro* and to examine the contribution of each individual RBD to this interaction, we expressed the wild-type and activated forms of H-Ras, Rap2A, and Rap2B in HEK 293T cells, and measured the RGS14/GTPase interaction using GST pull-down assays. Purified recombinant RGS14 (both full-length and truncated versions) interacted selectively with activated (and not wild-type) H-Ras, and this interaction was dependent upon the presence of the first RBD of RGS14 (Figure 1A; *e.g.*, compare GST-RGS14.RBD1 *vs* GST-RGS14.RBD2). We next examined the ability of GST-RGS14 fusion proteins to interact with wild-type and activated Rap2A and Rap2B. Interactions were observed with both Rap2A and Rap2B, and this binding appeared to be mediated by the first RBD in the tandem array; however, in contrast to the interaction with H-Ras (Figure 1A), the interaction was *independent* of the nucleotide state of Rap2A/2B (Figure 1B and 1C). (Note that endogenous RapGEF activity in HEK 293T cells could result in a significant amount of wild type Rap protein being GTP-bound.)

**Figure 2:**
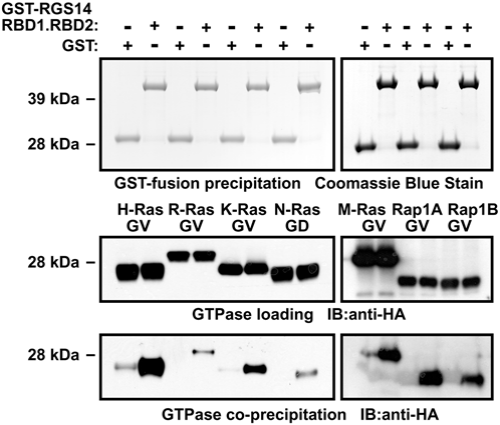
The Ras binding domains of RGS14 have promiscuous small GTPase selectivity *in vitro*. GST or a GST-fusion protein of RGS14(RBD1.RBD2) were incubated with lysates from HEK293T cells transfected with mutationally activated Ras-family GTPases. Protein complexes were precipitated with glutathione agarose, washed, and resolved by SDS-PAGE and immunoblot (IB) (bottom panels). Experimental samples were also analyzed by immunoblot to ensure equivalent loading of GTPases (middle panels). Precipitation of GST-fusion proteins was confirmed by SDS-PAGE and Coomassie Blue staining (top panels). Data are representative of 3 or more independent experiments.

As other Ras family members can interact with RBD-containing proteins [32], [33], we conducted a broader analysis of RGS14 selectivity for Ras family GTPases, initially in this *in vitro* setting with recombinant RGS14 protein. GST-RGS14(RBD1.RBD2) fusion protein interacted with activated versions of other Ras isoforms (K- and N-Ras) and R-Ras proteins (R-Ras1 and R-Ras3/M-Ras) in GST pull-down assays, suggesting that RGS14 is also capable of binding multiple Ras and R-Ras isoforms *in vitro* (Figure 2). Similarly, we examined the ability of RGS14 to interact with additional Rap isoforms. GST-RGS14(RBD1.RBD2) co-precipitated with activated Rap1A and Rap1B (Figure 2).

Isothermal titration calorimetry was then employed to demonstrate a direct *in vitro* protein-protein interaction and to measure the affinity of GDP or GPPNHP loaded H-Ras for RGS14(RBD1.RBD2). H-Ras was observed to directly interact with RGS14(RBD1.RBD2) in a 1-to-1 stoichiometry (Table 1 and Figure S1). The affinity between GTP-analogue bound (activated) H-Ras and RGS14(RBD1.RBD2) was significantly higher (K_D_ ∼10 µM) than that of GDP-bound (inactive) H-Ras and RGS14(RBD1.RBD2) (K_D_>200 µM) (Table 1).

**Table 1:** Thermodynamic parameters of H-Ras binding to the tandem Ras-binding domain region of RGS14 as measured by isothermal titration calorimetry.

Nucleotide	N^a^	K_A_ (M^−1^)	ΔH (kcal M^−1^)	ΔS (cal M^−1^K^−1^)	ΔG (kcal M^−1^)
GPPNHP	1.03±0.02	1.01×10^5^±7.4×10^3^	−1.81±0.04	16.6	−2.13
GDP	1.05±0.06	2.35×10^4^±1.9×10^3^	−7.1±0.5	−4.43	−7.17

a Thermodynamic parameters are stoichiometry (N), association constant (K_A_), enthalpy (ΔH), entropy (ΔS), and free energy (ΔG). Data are representative of 3 or more independent experiments.

### RGS14 preferentially interacts with activated H-Ras in cells

**Figure 3:**
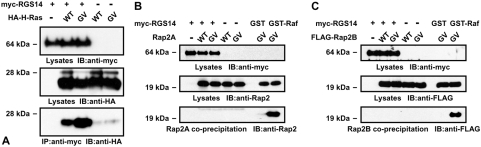
Full-length RGS14 interacts with activated H-Ras, but not Rap2A/B, in cells. HEK293T cells were transfected with plasmids encoding full-length, myc-epitope tagged RGS14, and wild-type (WT) or activated (GV), HA-tagged H-Ras (A), untagged Rap2A (B), or FLAG-tagged Rap2B (C). Cell lysates were immunoprecipitated (IP) with anti-myc antibodies or precipitated with GST or GST-Raf-1 (as controls). Total lysates and precipitates were immunoblotted (IB) with indicated antibodies. Data are representative of 3 or more independent experiments.

We examined the capacity of RGS14 to interact with Ras proteins in mammalian cell co-immunoprecipitation (co-IP) assays. Whereas the *in vitro* GST pull-down assays revealed promiscuous association of full-length RGS14 (and truncated forms containing the RBDs) with multiple different Ras isoforms (Figure 1 and Figure 2), in cells full-length RGS14 stably associated preferentially with activated H-Ras over other Ras isoforms (Figure 3A and Figure S2). We consistently observed cellular co-IP of full-length RGS14 with N-Ras(G12D), but it was of lower magnitude than binding to H-Ras(G12V) (Figure S2). Interestingly, we did not observe cellular co-IP between full-length RGS14 and Rap1A, Rap1B, Rap2A, nor Rap2B (Figures 3B, 3C, and S2), suggesting that the physiological Ras protein family target for RGS14 is H-/N-Ras, and not Rap GTPases. We also did not observe an interaction between RGS14 and activated Ran, Rab1, Arf1, Cdc42, RalA, RhoA, Rac1, nor Rac2 using cellular co-immunoprecipitation (Figure S2).

**Figure 4:**
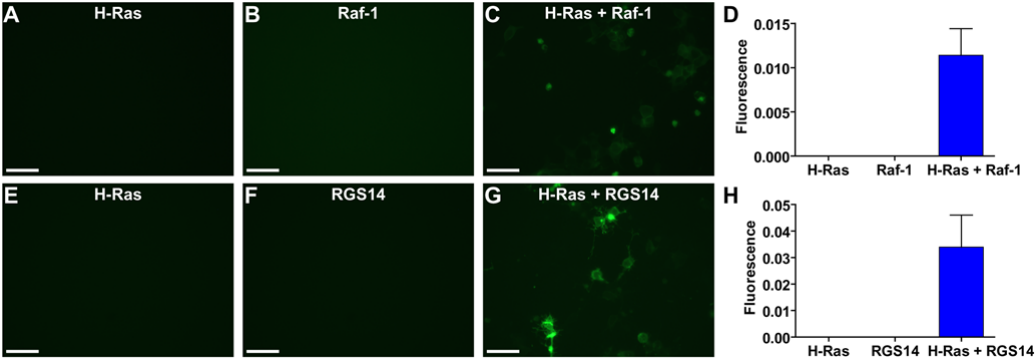
Full-length RGS14 and H-Ras interact in live cells. HEK293T cells were transfected with the indicated combinations of plasmids, encoding the N-terminal fragment of Yellow Fluorescent Protein (YFP) fused to the N-terminus of H-Ras(G12S), and the C-terminal fragment of YFP fused to the C-terminal of full-length Raf-1 or full-length RGS14, respectively. 48 h after transfection cells were analyzed by epifluorescence microscopy, and fluorescence was quantified using image analysis (as described in *Materials and Methods*). (A–D) Experiments measuring fluorescence complementation between H-Ras and Raf-1. (E–H) Experiments measuring fluorescence complementation between H-Ras and RGS14. Scale bars represent 50 µm. Data are representative of 3 or more independent experiments. Additional control experiments are presented in Figure S3.

To examine whether full-length RGS14 and activated H-Ras form a stable complex in cells, we used yellow fluorescent protein (YFP) bimolecular fluorescence complementation [34], [35]. DNA encoding N-terminal (YFP_N_) and C-terminal fragments (YFP_C_) of YFP were cloned in-frame with target proteins. As a positive control, we first examined the ability of YFP_N_-H-Ras(G12S) and YFP_C_-Raf-1 to reconstitute YFP fluorescence [36]. Cellular expression of YFP_N_-H-Ras(G12S) alone or YFP_C_-Raf-1 alone did not produce fluorescence (Figure 4A,B,D); however, co-expression of both proteins resulted in fluorescence complementation (Figure 4C,D). Next, we expressed YFP_N_-H-Ras(G12S) alone, YFP_C_-RGS14 alone, or YFP_N_-H-Ras(G12S) and YFP_C_-RGS14, and examined reconstitution of YFP. When expressed individually, H-Ras(G12S) and full-length RGS14 did not produce measurable fluorescence (Figure 4E,F,H); however, when co-expressed, the fluorescence intensity was substantially increased (Figure 4G,H), thus demonstrating that H-Ras and RGS14 interact in live cells. We performed a comprehensive panel of positive and negative control experiments using various YFP_N_ and YFP_C_ fusion proteins (Figure S3). These controls demonstrate the high efficiency and specificity of YFP fluorescence complementation induced by interaction between H-Ras(G12S) and RGS14. It is of note that YFP_C_-RGS14 complemented YFP_N_-H-Ras(G12S) with better efficiency than did YFP_C_-Raf-1 (Figure 4 and Figure S3), and with comparable efficiency to the constitutive heterodimer of YFP_C_-Gβ_1_ and YFP_N_-Gγ_2_ (Figure S3).

### RGS14 coordinates an activated H-Ras-dependent B-Raf/MEK1/ERK1 complex

**Figure 5:**
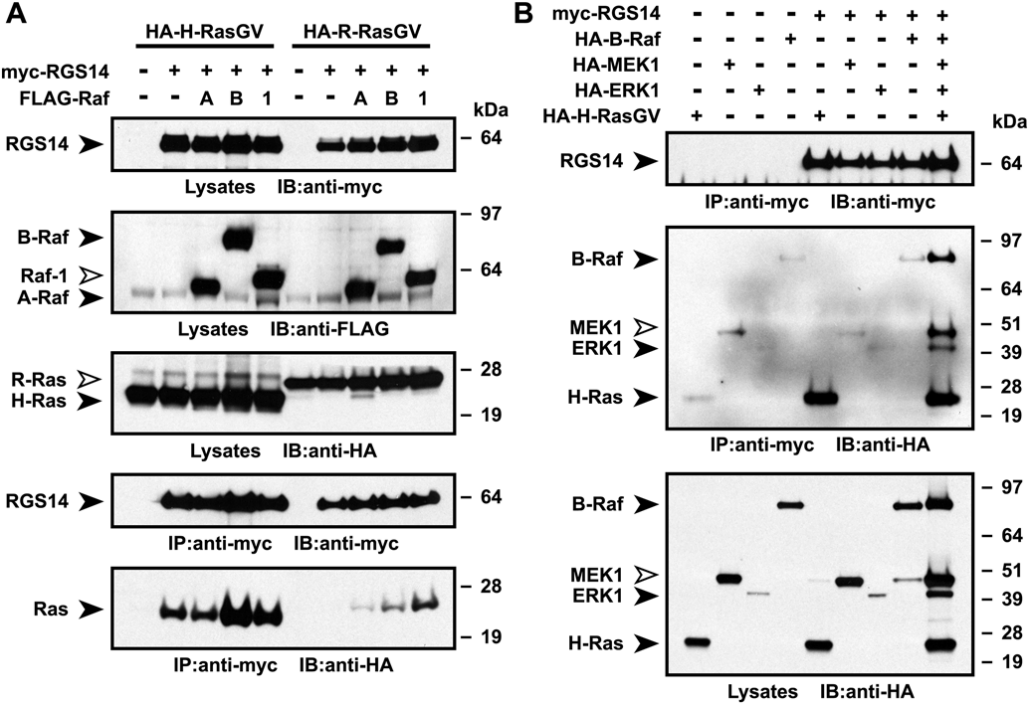
Full-length RGS14 forms a multiprotein complex with ERK MAPK pathway components dependent on activated H-Ras. (A) HA-tagged, activated H-Ras(G12V) or R-Ras(G38V) was co-transfected with empty vector, full-length myc-RGS14, or with full-length myc-RGS14 and FLAG-tagged A-Raf (“*A*”), B-Raf (“*B*”), or Raf-1 (“*1*”) expression vectors in HEK293T cells. Cell lysates were immunoprecipitated (IP) with anti-myc antibodies. Total lysates and immunoprecipitates were immunoblotted (IB) with indicated antibodies. (B) HEK293T cells were transfected with plasmids encoding full-length myc-RGS14, HA-H-Ras(G12V), HA-B-Raf, HA-MEK1, and HA-ERK1 in various combinations as indicated. Cell lysates were immunoprecipitated (IP) with anti-myc antibodies. Total lysates and immunoprecipitates were immunoblotted (IB) with indicated antibodies. Data are representative of 3 or more independent experiments.

To investigate the interaction of RGS14 with multiple components of the Ras-ERK MAPK signaling pathway, we co-expressed RGS14 and activated Ras GTPases with Raf kinase isoforms A-Raf, B-Raf, or Raf-1, and examined the ability of RGS14 to bind to Ras. Full-length RGS14 does not interact with activated R-Ras in cells (Figure 5A and Figure S2); however, both activated H-Ras and R-Ras interact with all three Raf isoforms (data not shown; reviewed in [37]). Activated R-Ras did not co-immunoprecipitate with RGS14 in the absence of any of the three Raf kinases (Figure 5A); however, in the presence of the three Raf kinases, we observed weak interactions with R-Ras (Figure 5A) that were comparable to the preference of R-Ras for each of the three Raf isoforms (data not shown). In contrast, the amount of H-Ras bound to RGS14 dramatically increased upon concomitant expression of B-Raf and Raf-1, but not A-Raf (Figure 5A), consistent to our previous observations of cooperative binding with the related protein RGS12 [20]. This interaction was specific and not an artifact of non-specific binding of the complex to beads (Figure S4).

We also examined whether RGS14 was able to individually or simultaneously interact with multiple ERK MAPK components in cells. Activated H-Ras was detected in RGS14 immunoprecipitates upon their co-expression (Figure 5B and also Figure 3A). In contrast, we did not observe binary interactions between RGS14 and B-Raf, MEK1, nor ERK1, respectively (Figure 5B). However, when RGS14 was co-expressed with activated H-Ras, B-Raf, MEK1, and ERK1, we isolated a complex containing all five proteins (Figure 5B).

### Loss of RGS14 inhibits NGF-mediated neurite outgrowth in PC12 cells

Stimulation of the NGF receptor, TrkA, causes terminal differentiation, growth inhibition, and neurite formation in PC12 cells [38], [39]. NGF induces rapid and sustained activation of both Ras and ERK, and inhibition of either Ras or ERK blocks neurite induction [40]. Thus, NGF-induced neurite formation is mediated by Ras activation of the ERK MAPK cascade. Loss of RGS12 (a paralogue of RGS14) leads to reduction in NGF-promoted neurite outgrowth of PC12 cells [20]; thus, we hypothesized that RGS14 may also play an important role in neuritogenesis in PC12 cells.

**Figure 6:**
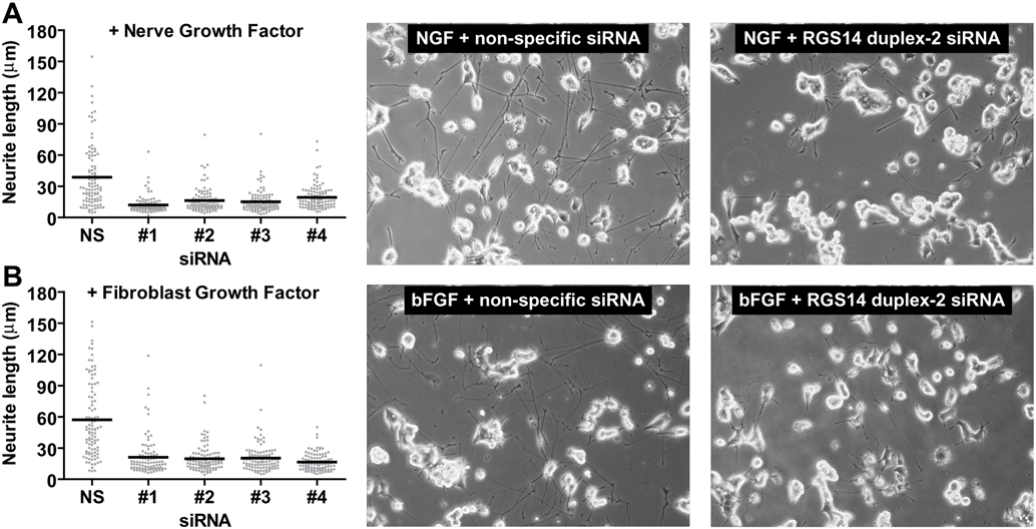
RGS14 knockdown inhibits NGF- and bFGF-induced neurite outgrowth. PC12 cells were transfected with a control non-specific (NS) siRNA or four independent RGS14 siRNA duplexes (#1-4). Twenty four hours later, cells were treated with 100 ng/ml NGF (A) or 100 ng/ml bFGF (B). Forty eight hours after growth factor treatment, neurite outgrowth was visualized by phase contrast microscopy and digital image capture. Representative images are presented to illustrate the effect of non-specific *versus* RGS14 duplex-2 siRNA on neurite outgrowth (right panels). The length of >100 neurites per condition was quantifed using ImageJ. Data are plotted as dot plots with the mean of each condition represented by a black line. Statistical significance was assessed by Kruskal-Wallis test with Dunn's post test. For both NGF- and bFGF-stimulated neurite outgrowth, statistical significance (P<0.001) was obtained for NS *versus* siRNA duplexes #1, #2, #3, and #4. Data are representative of 3 or more independent experiments.

**Figure 7:**
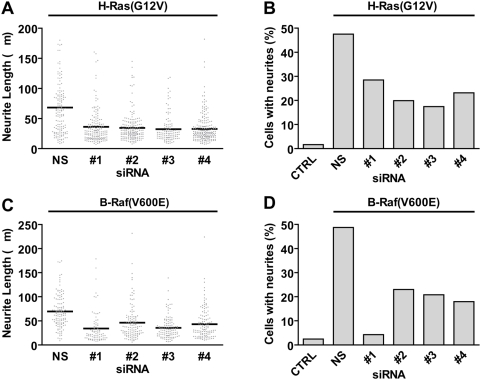
RGS14 knockdown inhibits activated H-Ras- and activated B-Raf-induced neurite outgrowth. PC12 cells were co-transfected with expression vectors for constitutively-activated H-Ras(G12V) (A, B) or B-Raf(V600E) (C, D) with either a non-specific (NS) siRNA duplex or one of four independent RGS14 siRNA duplexes (#1-4). Cells left untransfected are denoted control (CTRL). Seventy-two hours after transfection, neurite outgrowth was visualized by phase contrast microscopy and digital image capture. The length of >82 neurites per condition was quantifed using ImageJ. Data are plotted as dot plots with the mean of each condition represented by a black line (A, C). Significance was assessed by Kruskal-Wallis test with Dunn's post test. For both B-Raf(V600E)- and H-Ras(G12V)-stimulated neurite outgrowth, P<0.001 for NS *versus* #1, #2, #3, and #4. (B, D) The percentage of cells with neurites longer than one cell body length was measured for the experiments presented in panels A and C. Data are representative of 3 or more independent experiments.

To address a possible role for RGS14 in neurite formation, we employed rat RGS14 directed-siRNA to suppress endogenous RGS14 expression. A pool of four individual duplexes efficiently reduced RGS14 expression at both the protein (Figure S5A) and mRNA levels (Figure S6B). Upon their separation, all four individual oligonucleotide duplexes also were found to efficiently knockdown expression of RGS14 (Figure S5B and Figure S6B). The RGS14-directed siRNAs did not silence RGS12 expression in PC12 cells (Figure S6A), thus demonstrating the specific nature of these reagents. RNAi-induced reduction of RGS14 expression impaired NGF-mediated neurite formation when compared to cells treated with control siRNA (Figure 6A); this led to a significant reduction in the average length of NGF-promoted neurites compared to cells transfected with non-specific siRNA (Figure 6A). bFGF can reproduce the entire spectrum of PC12 cell responses known to be elicited by NGF, including neurite outgrowth [41]; thus, we also examined whether bFGF-promoted neurite outgrowth is affected by RGS14 suppression. Suppression of RGS14 also blocked neuritogenesis promoted by bFGF compared to cells transfected with non-specific siRNA (Figure 6B). To further establish the role of RGS14 in MAPK cascade-dependent neuritogenesis, we also examined the effect of RGS14 knockdown on PC12 neurite outgrowth stimulated by activated mutants of H-Ras (G12V; ref. [42]) and B-Raf (V600E; ref. [43]). Knockdown of RGS14 impaired both H-Ras- and B-Raf-stimulated neurite formation (Figure 7).

### Sustained activation of ERK by NGF and bFGF is reduced upon knockdown of RGS14

**Figure 8:**
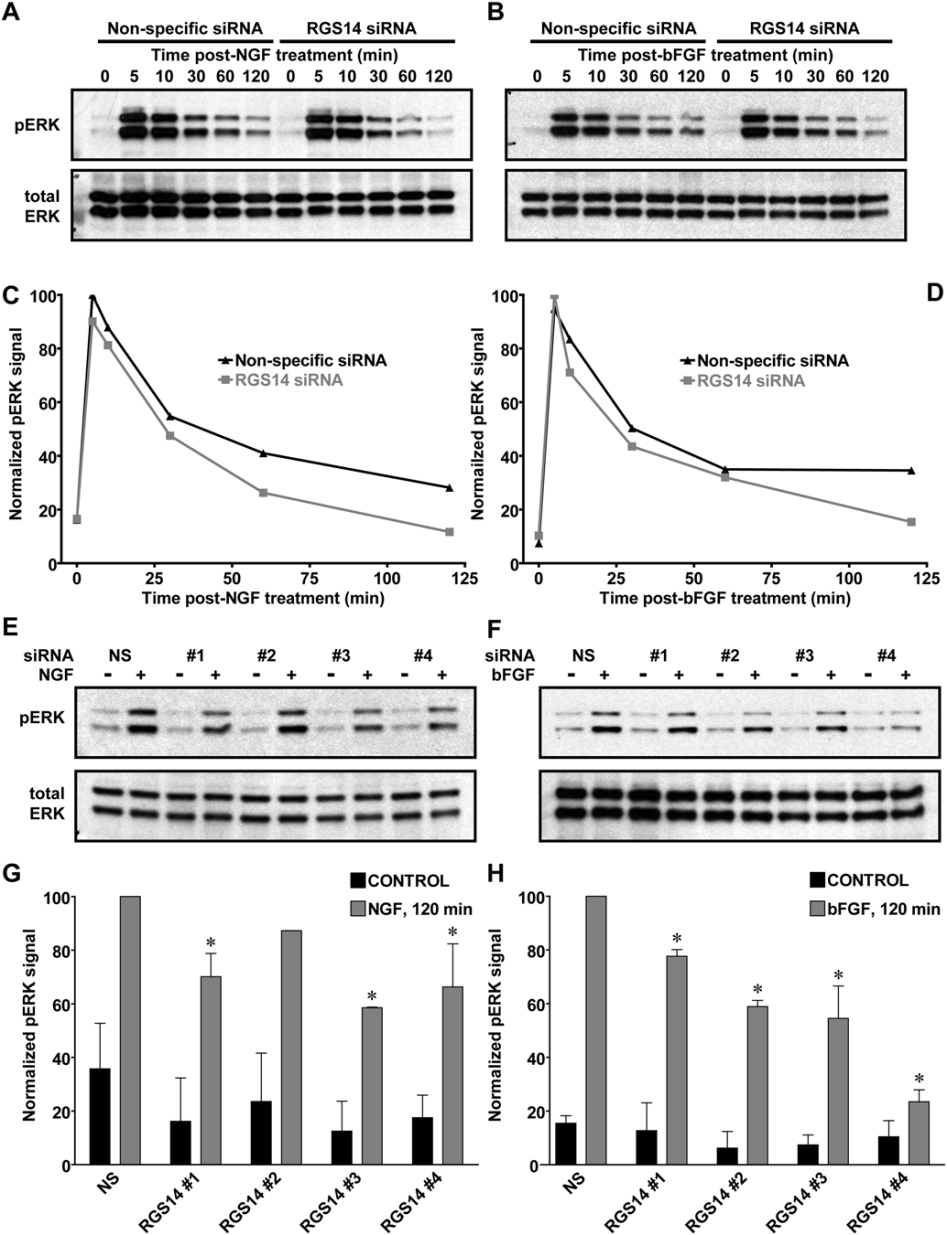
NGF- and bFGF-stimulated prolonged ERK activation is attenuated by RGS14 knockdown. (A, B) PC12 cells were transfected with control non-specific or pooled RGS14 siRNAs. Twenty four hours later, cells were stimulated with (A) NGF (100 ng/ml) or (B) bFGF (100 ng/ml). At indicated time points, cells were lysed and then subsequently analyzed by immunoblot for both phosphorylated, activated ERK1/2 (pERK) and total ERK1/2. Data are representative of 3 or more independent experiments. (C, D) Data from panels A and B were quantified using densitometry. (E, F) PC12 cells were transfected with control non-specific (NS) or 4 independent RGS14 (#1, #2, #3, #4) siRNA duplexes. Twenty four hours later, cells were stimulated with (E) NGF (100 ng/ml) or (F) bFGF (100 ng/ml). After 120 minutes, cells were lysed and analyzed by immunoblot for both phosphorylated, activated ERK1/2 (pERK) and total ERK1/2. (G, H) Data from multiple experiments (n = 2 to 4) conducted as described in panels E and F were quantified using densitometry. Statistical significance was determined using ANOVA with Dunnett's multiple comparison test (* denotes P<0.05 *vs* NS siRNA samples [n = 3]). For the NGF-treated samples in panel E, the decrease in phospho-ERK level at the 120 min time-point in RGS14 siRNA #2 treated cells was not tested for statistical signficiance, given that the sample set for the RGS14 siRNA#2 treatment was n = 2.

In PC12 cells, sustained ERK activation promotes cell differentiation, whereas a more transient duration of ERK activation promotes growth [44]–[46]. Specifically, NGF, acting through the TrkA receptor, induces both transient and prolonged activation of ERK, with the prolonged activation required for neuritogenesis [46], [47]. To examine the effect of RGS14 knockdown on ERK activation, PC12 cells were transfected with either non-specific siRNA or a pool of four RGS14 siRNA duplexes (Figure 8A), and stimulated with NGF or bFGF. We observed a reduction in the duration of ERK activation upon RGS14 depletion when compared to cells transfected with non-specific siRNA (Figure 8A–D). Next, we examined whether the individual oligonucleotides were capable of reducing prolonged ERK activation by NGF and bFGF. The duration of ERK activation by NGF and bFGF was shortened by RGS14 knockdown (Figure 8E–H).

## Discussion

Our present study has generated the following major findings: (i) although RGS14 interacts with a wide array of Ras and Rap isoforms *in vitro*, the most likely cellular target for full-length RGS14 is activated H-Ras; (ii) the binding of activated H-Ras to RGS14 facilitates assembly of a multiprotein complex with components of the ERK MAPK cascade (B-Raf, MEK1, and ERK1); (iii) loss of RGS14 expression blunts both NGF- and bFGF-promoted neurite outgrowth of PC12 cells; and (iv) duration of ERK activation by NGF and bFGF is shortened by RGS14 knockdown, suggesting a mechanistic explanation for impairment of agonist-promoted neuritogenesis seen upon RGS14 depletion. Our findings are in contrast to the original yeast two-hybrid analysis of interactions between RGS14 and Ras-family GTPases described by Traver *et al.* [11], in which interaction between RGS14 and activated Rap1B, but not H-Ras, was observed. It is important to note that we have independently replicated the yeast-based data of Traver *et al.* [11] using (as bait) the tandem RBD C-terminal portion of RGS14 (Figure S7). This discrepancy between yeast two-hybrid and *in vitro*/cellular experiments highlights the importance of examining protein-protein interactions under a variety of experimental conditions.

Our demonstration that recombinant RGS14 (both full-length and truncated species) can bind promiscuously to multiple Ras- and Rap-family GTPases *in vitro* is not surprising as the switch regions of Ras-family GTPases, which participate in the interactions with Ras-binding domains, are highly conserved [48]. Yet, despite reports claiming RGS14 as a putative Rap effector [11], [18], we were unable to demonstrate interaction between Rap and RGS14 in a mammalian cellular environment. We are unable to explain why the yeast two-hybrid system demonstrates that Rap1B, but not H-Ras, interacts with the RGS14 RBD region (ref. [11] and Figure S7). This suggests that, although it is a powerful discovery technique, the yeast two-hybrid system should not be used in isolation to draw conclusions about *in vivo* protein-protein interaction specificity. Indeed, it has been estimated that over 50% of reported yeast two-hybrid interactions are false positives [49].

Traver *et al.* also used purified proteins and were unable to detect an interaction between H-Ras and RGS14 [11]; we are unable to explain this difference with our present work, although we note that another group has demonstrated that H-Ras can bind to RGS14 *in vitro* [16]. We also note that Traver *et al.* may have been using low-sensitivity detection methods, as they were not able to observe interaction between RGS14 and Gα_i1_/Gα_i3_ [11], the latter proteins being well-established, nanomolar affinity interaction partners of the RGS14 C-terminal GoLoco motif [14]. Although we did not observe an interaction between RGS14 and Rap isoforms in cells, we have not definitively ruled out that these proteins interact *in vivo*. It may be that post-translational modification of RGS14 or Rap directly influences Rap/RGS14 interaction or directs these proteins to a distinct subcellular locale that facilitates their subsequent interaction [50], [51]. Our data demonstrate that RBD1 is the binding site for activated monomeric GTPases in RGS14. This is concordant with *in vitro* and yeast two-hybrid experiments [17], [18]. RBD2 within RGS12 appears to be involved in recruiting Raf to form a MAPK scaffolding complex, as a loss-of-function mutation within RBD1 inhibits the RGS12/H-Ras interaction, but not the RGS12/B-Raf association [20]. We speculate that RBD2 may possess the same function within RGS14.

Our observations as to the cellular selectivity of RGS14 are intriguing, in that we demonstrated that RGS14 can interact with H-Ras and, to a lesser extent, with N-Ras. Despite extensive studies, the *in vivo* mechanisms of Ras-effector GTPase selectivity are still not fully defined [52]. One contribution to *in vivo* selectivity is likely differential subcellular localizations of these GTPases, arising from post-translational modifications and/or unique hypervariable linker domain sequences outside the effector domains of Ras family members. Additionally, regions beyond the RBDs of RGS14, *e.g.*, the RGS domain and GoLoco motif, may play a role in the selectivity of RGS14 for activated H-Ras in cells.

The apparent affinity of activated GTPases for the tandem RBD region of RGS14 *in vitro* is weak (*e.g.*, for H-Ras·GPPNHP, K_D_ = 10 µM); it is thus most likely that other determinants and protein partners facilitate the formation of high affinity complexes *in vivo*. Despite being unable to observe binary interactions between RGS14/B-Raf, RGS14/MEK1, or RGS14/ERK1, RGS14 appears to assemble a stable, multiprotein complex containing H-Ras, B-Raf, MEK1, and ERK1 when all five proteins are expressed concomitantly (Figure 5). One report has asserted that Rap2A is unable to modulate the Gα-directed GAP or GDI activities of RGS14 *in vitro* [17]. However, these experiments were conducted using protein concentrations of Rap2A and RGS14 that are orders of magnitude below the determined K_D_ values [17]. Thus it remains to be determined whether Ras-family GTPase binding to RGS14 can modulate the GAP and GDI functions of this molecule.

Our present findings with RGS14, in combination with our previous work on RGS12 [20], support the notion that both RGS proteins can function to organize multiprotein complexes containing Ras/Raf/MEK/ERK; however, how these two RGS proteins achieve this function appears different. Firstly, RGS14 does not appear to bind directly to Raf, MEK, or ERK; in contrast, RGS12 binds directly to both B-Raf and MEK2 [20]. This distinction most likely arises from the unique domain architecture of RGS12, which contains two additional domains (N-terminal PDZ and PTB domains) not present in RGS14. We established that RGS12 binds to MEK2 via its PDZ domain, and B-Raf via its tandem RBDs [20]. As RGS14 also contains tandem RBDs, it is surprising that RGS14 does not bind directly to B-Raf. Our present data suggest that RGS14 most likely assembles a MAPK multienzyme complex differently than RGS12. This highlights the possibility that RGS14 might require additional protein partners beyond the MAPK members organized in the complex. Such a requirement for additional accessory proteins would increase the complexity of possible signaling cascades that are regulated by RGS14; it is within this scenario that RGS14 may interact with and modulate Rap-mediated signaling.

Secondly, knockdown of RGS14 in PC12 cells inhibits both NGF- and bFGF-mediated neuritogenesis, whereas depletion of RGS12 selectively inhibits only NGF-promoted neuronal differentiation. This selective modulation of growth factor receptor signaling may be due, at least in part, to the ability of RGS12 to bind to the NGF receptor TrkA, but not to FGFR1 [20]. While we have shown that (a) RGS12 associates with TrkA, (b) RGS12 undergoes subcellular redistribution in response to NGF stimulation, and (c) RGS12 is localized coincident with endosomal markers in cells, we presently have no evidence for any of these functions or behaviors for RGS14. In contrast, RGS14 is typically localized to the cytosol, nucleus, and perinuclear regions in interphase, and on microtubule structures during mitosis [53]–[55]. Thus, coordinating activated Ras and the MAPK cascade at subcellular locales distinct from RGS12 likely engenders a different set of outputs (*i.e.*, distinct ERK phosphorylation substrates) from RGS14-dependent signaling; independent MAPK signaling dependent on RGS14 that is equally critical for an integrated, long-term phenotypic response to a growth factor like NGF would explain why RGS12 is not able to compensate for the loss of RGS14 in NGF-induced neuritogenesis in siRNA-treated PC12 cells.

It is important to note also that RGS14 has biochemical properties atypical of a classical MAPK scaffold such as RGS12, MP1, STE5, and others. We were unable to detect binary interaction of RGS14 with any MAPK pathway members other than Ras. Typical MAPK scaffolds demonstrate binary interactions with multiple MAPK components. It is possible that H-Ras binding induces a conformational change in RGS14 that facilitates binding to additional MAPK pathway members, or that interaction with MAPK members is activation-dependent. Cellular evidence for a MAPK scaffolding-like function for RGS14 is provided by the requirement of RGS14 expression for B-Raf(V600E)-induced signal transduction in PC12 cells (Figure 7). We have not yet delineated the structural determinants of multiprotein-complex formation between Ras, RGS14, and Raf. We hypothesize that this interaction is unlikely due to simultaneous binding of RGS14 and Raf to activated H-Ras. Both RBD1 of RGS14 and the sole RBD of Raf proteins represent evolutionarily conserved binding sites for the effector loops (switch regions) of activated Ras-family GTPases [16], [56]. Thus, based on the current structural knowledge, simultaneous binding of a single molecule of activated H-Ras to two RBDs is highly improbable. Evidence supporting the alternative view of Ras/RGS14/Raf complex formation is illustrated in Figure 5A, as formation of this complex is Raf-isoform selective. In the absence of RGS14, H-Ras(G12V) interacts equivalently with Raf-1, A-Raf, and B-Raf (data not shown). However, in the presence of RGS14, complex formation showed distinct selectivity towards B-Raf and Raf-1, but not A-Raf, in terms of the amount of H-Ras(G12V) co-precipitated. This suggests that facile co-precipitation of Ras in binary complexes with RGS14 and with Raf is not occurring and that a Raf-isoform selective phenomenon is being observed.

In conclusion, our studies delineate a potential major difference between the physiological roles of RGS12 and RGS14. Conventional MAPK scaffold proteins execute two main functions: (i) tethering proteins together, and (ii) specifying the subcellular localization of the multiprotein complex which, in turn, guides their final output. The finding that RGS12 is localized to endocytic vesicles and acts as a conventional MAPK scaffold that regulates NGF-promoted signaling in both PC12 and DRG neurons [20] supports the notion that RGS12 and its partners are key components of ‘signaling endosomes’ that form in the axon terminal and traffic in a retrograde manner to the cell body where they initiate local signal transduction cascades [57]. Thus, the subcellular localization of RGS14 is distinct from that of RGS12, and this may be reflective of functional differences in the ability to modulate signal transduction, such as the ability of RGS14, but not RGS12, to modulate FGFR-mediated signal transduction. Thus, it is likely that, *in vivo*, RGS14 integrates signaling independent of, and with different consequences than, RGS12.

## Supporting Information

Figure S1 Guanine nucleotide-state selective interaction between H-Ras and RGS14. Isothermal titration calorimetry was used to measure the interaction between H Ras and the isolated Ras-binding domains of RGS14 (“RGS14(RBD1.RBD2)”). A stepwise titration of 300 µM RGS14(RBD1.RBD2) protein into a cell containing 20 µM H Ras(GPPNHP) (A) or H-Ras(GDP) (B) was performed and the heat change accompanying RGS14 injection was detected by comparison with a reference cell. RGS14(RBD1.RBD2) injected into buffer alone was used as a negative control. Heat changes were plotted against the molar ratio of H Ras to RGS14(RBD1.RBD2) protein and analyzed using non-linear regression (see Table 1 of the main manuscript for data analysis parameters). Data was fit by applying a one-site binding model involving exothermic reaction phases (negative enthalpy changes) with favorable free energy changes. Analysis of the data indicates that complete saturation of the binding site is not achieved. This is likely due to the high dissociation rate of the complex.
(0.66 MB TIF)

Figure S2 RGS14 selectively interacts with H-Ras and not other small GTPases in cells. HEK293T cells were transfected with plasmids encoding full-length myc-RGS14 and various mutationally-activated (“GV”, “GD”, or “QL”), HA-epitope tagged GTPases. Cell lysates were immunoprecipitated (IP) with anti-myc antibodies. Total lysates and precipitates were immunoblotted (IB) with indicated antibodies. “Arf GTPase” denotes the use of Arf1.
(3.18 MB TIF)

Figure S3 Specificity of fluorescence complementation between H-Ras(G12S) and RGS14. HEK293T cells were co-transfected with cDNAs encoding the empty vector pcDNA3.1, the N-terminal (amino acids 1–159) and C-terminal (amino acids 159-239) fragments of Yellow Fluorescent Protein (YFPN and YFPC), and indicated proteins fused to YFPN and YFPC. 48 hours after transfection, cells were analyzed by epifluorescence microscopy, and fluorescence was quantified using image analysis as described in the Experimental section. (A) Transfection of the YFPC vector or YFPC-fusion constructs does not result in measurable fluorescence in the absence of YFPN co-transfection. (B) YFPN alone does not complement YFPC nor YFPC-fusion constructs. (C) YFPN-H-Ras(G12S) complements both RGS14-YFPC and Raf-1-YFPC but not YFPC nor YFPC-Gβ1. (D) YFPN-Gγ2 complements YFPC-Gβ1 but not YFPC, RGS14-YFPC, nor Raf-1-YFPC.
(0.86 MB TIF)

Figure S4 Specificity of H-Ras/RGS14/B-Raf complex formation. HEK293T cells were transfected with plasmids encoding full-length myc-RGS14, HA-B-Raf, and either wild-type or G12V HA-H-Ras. Cell lysates were immunoprecipitated (IP) with anti-myc antibodies and protein A/G agarose, as indicated. Total lysates and immunoprecipitates were immunoblotted (IB) with indicated antibodies.
(3.08 MB TIF)

Figure S5 Specificity and efficacy of rat RGS14 siRNAs (I). (A) HEK293T cells were transfected with HA-epitope tagged RGS14 expression vector and then 6 hours later transfected with control non-specific (NS) siRNA or a pool of four RGS14 siRNAs. 24, 48, and 72 hours later, RGS14 expression level was analyzed by immunoblot (IB) with anti-HA. Samples were immunoblotted with anti-actin antibodies as a control for total protein levels. (B) HEK293T cells were transfected with myc-epitope tagged RGS14 expression vector and then 6 hours later transfected with control non-specific (NS) siRNA or four independent RGS14 siRNA duplexes (#1-4) that constitute the siRNA SMARTpool used in panel A. 48 hours later RGS14 expression level was analyzed by immunoblot with anti-myc antibodies. Samples were immunoblotted with anti-actin antibodies as a control for total protein levels.
(1.89 MB TIF)

Figure S6 Specificity and efficacy of rat RGS12 and RGS14 siRNAs (II). PC-12 cells were transfected with control non-specific (NS) siRNA, RGS12(D2) siRNA (duplex 2 from ref. 20), a SMARTpool (SP) of four RGS14 siRNA duplexes, or the four individual constituent siRNA duplexes (D1, D2, D3, and D4) which comprise the SMARTpool. Forty eight hours later, cells were harvested, RNA was extracted, and RGS12 (A) and RGS14 (B) expression levels were measured by quantitative real-time PCR (as performed by the Gene Expression Core of the UNC Dept. of Laboratory Medicine and Pathology, directed by Dr. Hyung-Suk Kim). RGS12 and RGS14 data were normalized for relative expression levels using the 2-(<delta><delta>Ct) method with β-actin as the internal control. Data is presented as relative expression compared to non-specific (NS) siRNA treated samples. Statistical significance was determined using ANOVA with Dunnett's multiple comparison test (* denotes P<0.5 vs NS siRNA samples).
(0.30 MB TIF)

Figure S7 Yeast two-hybrid analysis of interactions between RGS14 and Ras-family GTPases. Yeast were co-transformed with bait plasmids encoding indicated GTPase fusions with the Gal4p DNA binding domain and prey plasmids encoding either Raf-1 or RGS14 fused to the Gal4p activation domain. Wild-type (WT) or glycine-12-to-valine (GV) mutationally-activated GTPases were used to test for activation-dependent binding to the Ras-binding domain (RBD) of Raf-1 (amino acids 50–131) and the tandem RBDs and GoLoco motif of RGS14 (amino acids 263–544). Yeast were plated on synthetic defined agar (SDA), lacking leucine (-Leu, to select for the pACT-II plasmid containing the LEU2 gene), and tryptophan (-Trp, to select for the pGBT9 plasmid containing the TRP1 gene). Growth on SDA-Leu-Trp demonstrates incorporation of bait and prey plasmids (top panel). Growth on SDA-Leu-Trp-His in the presence of the histidine biosynthesis inhibitor 3-amino-1,2,4-triazole (3AT) indicates a positive protein-protein interaction.
(2.04 MB TIF)

Table S1 DNA constructs created and obtained for use in this study.
(0.10 MB PDF)

## References

[1] Cabrera-Vera TM, Vanhauwe J, Thomas TO, Medkova M, Preininger A (2003). Insights into G protein structure, function, and regulation.. Endocr Rev.

[2] McCudden CR, Hains MD, Kimple RJ, Siderovski DP, Willard FS (2005). G-protein signaling: back to the future.. Cell Mol Life Sci.

[3] Pierce KL, Premont RT, Lefkowitz RJ (2002). Seven-transmembrane receptors.. Nat Rev Mol Cell Biol.

[4] Siderovski DP, Willard FS (2005). The GAPs, GEFs, and GDIs of heterotrimeric G-protein alpha subunits.. Int J Biol Sci.

[5] Soundararajan M, Willard FS, Kimple AJ, Turnbull AP, Ball LJ (2008). Structural diversity in the RGS domain and its interaction with heterotrimeric G protein alpha-subunits.. Proc Natl Acad Sci U S A.

[6] Tesmer JJ, Berman DM, Gilman AG, Sprang SR (1997). Structure of RGS4 bound to AlF4–activated G(i alpha1): stabilization of the transition state for GTP hydrolysis.. Cell.

[7] Berman DM, Wilkie TM, Gilman AG (1996). GAIP and RGS4 are GTPase-activating proteins for the Gi subfamily of G protein alpha subunits.. Cell.

[8] Ross EM, Wilkie TM (2000). GTPase-activating proteins for heterotrimeric G proteins: regulators of G protein signaling (RGS) and RGS-like proteins.. Annu Rev Biochem.

[9] De Vries L, Gist Farquhar M (1999). RGS proteins: more than just GAPs for heterotrimeric G proteins.. Trends Cell Biol.

[10] Snow BE, Antonio L, Suggs S, Gutstein HB, Siderovski DP (1997). Molecular cloning and expression analysis of rat Rgs12 and Rgs14.. Biochem Biophys Res Commun.

[11] Traver S, Bidot C, Spassky N, Baltauss T, De Tand MF (2000). RGS14 is a novel Rap effector that preferentially regulates the GTPase activity of galphao.. Biochem J.

[12] Cho H, Kozasa T, Takekoshi K, De Gunzburg J, Kehrl JH (2000). RGS14, a GTPase-activating protein for Gialpha, attenuates Gialpha- and G13alpha-mediated signaling pathways.. Mol Pharmacol.

[13] Hollinger S, Taylor JB, Goldman EH, Hepler JR (2001). RGS14 is a bifunctional regulator of Galphai/o activity that exists in multiple populations in brain.. J Neurochem.

[14] Kimple RJ, De Vries L, Tronchere H, Behe CI, Morris RA (2001). RGS12 and RGS14 GoLoco motifs are G alpha(i) interaction sites with guanine nucleotide dissociation inhibitor Activity.. J Biol Chem.

[15] Mittal V, Linder ME (2004). The RGS14 GoLoco domain discriminates among Galphai isoforms.. J Biol Chem.

[16] Kiel C, Wohlgemuth S, Rousseau F, Schymkowitz J, Ferkinghoff-Borg J (2005). Recognizing and defining true Ras binding domains II: in silico prediction based on homology modelling and energy calculations.. J Mol Biol.

[17] Mittal V, Linder ME (2006). Biochemical characterization of RGS14: RGS14 activity towards G-protein alpha subunits is independent of its binding to Rap2A.. Biochem J.

[18] Traver S, Splingard A, Gaudriault G, De Gunzburg J (2004). The RGS (regulator of G-protein signalling) and GoLoco domains of RGS14 co-operate to regulate Gi-mediated signalling.. Biochem J.

[19] Formstecher E, Aresta S, Collura V, Hamburger A, Meil A (2005). Protein interaction mapping: a Drosophila case study.. Genome Res.

[20] Willard MD, Willard FS, Li X, Cappell SD, Snider WD (2007). Selective role for RGS12 as a Ras/Raf/MEK scaffold in nerve growth factor-mediated differentiation.. Embo J.

[21] Hains MD, Siderovski DP, Harden TK (2004). Application of RGS box proteins to evaluate G-protein selectivity in receptor-promoted signaling.. Methods Enzymol.

[22] Kim HS, Lee G, John SW, Maeda N, Smithies O (2002). Molecular phenotyping for analyzing subtle genetic effects in mice: application to an angiotensinogen gene titration.. Proc Natl Acad Sci U S A.

[23] Livak KJ, Schmittgen TD (2001). Analysis of relative gene expression data using real-time quantitative PCR and the 2(-Delta Delta C(T)) Method.. Methods.

[24] Willard FS, Siderovski DP (2004). Purification and in vitro functional analysis of the Arabidopsis thaliana regulator of G-protein signaling-1.. Methods Enzymol.

[25] Soundararajan M, Yang X, Elkins JM, Sobott F, Doyle DA (2007). The centaurin gamma-1 GTPase-like domain functions as an NTPase.. Biochem J.

[26] Martin-McCaffrey L, Willard FS, Pajak A, Dagnino L, Siderovski DP (2005). RGS14 is a microtubule-associated protein.. Cell Cycle.

[27] Snow BE, Brothers GM, Siderovski DP (2002). Molecular cloning of regulators of G-protein signaling family members and characterization of binding specificity of RGS12 PDZ domain.. Methods Enzymol.

[28] James P, Halladay J, Craig EA (1996). Genomic libraries and a host strain designed for highly efficient two-hybrid selection in yeast.. Genetics.

[29] Peterson SN, Trabalzini L, Brtva TR, Fischer T, Altschuler DL (1996). Identification of a novel RalGDS-related protein as a candidate effector for Ras and Rap1.. J Biol Chem.

[30] Van Aelst L, Barr M, Marcus S, Polverino A, Wigler M (1993). Complex formation between RAS and RAF and other protein kinases.. Proc Natl Acad Sci U S A.

[31] Durfee T, Becherer K, Chen PL, Yeh SH, Yang Y (1993). The retinoblastoma protein associates with the protein phosphatase type 1 catalytic subunit.. Genes Dev.

[32] Rodriguez-Viciana P, Sabatier C, McCormick F (2004). Signaling specificity by Ras family GTPases is determined by the full spectrum of effectors they regulate.. Mol Cell Biol.

[33] Wennerberg K, Rossman KL, Der CJ (2005). The Ras superfamily at a glance.. J Cell Sci.

[34] Kerppola TK (2006). Visualization of molecular interactions by fluorescence complementation.. Nat Rev Mol Cell Biol.

[35] Remy I, Michnick SW (2004). Mapping biochemical networks with protein-fragment complementation assays.. Methods Mol Biol.

[36] MacDonald ML, Lamerdin J, Owens S, Keon BH, Bilter GK (2006). Identifying off-target effects and hidden phenotypes of drugs in human cells.. Nat Chem Biol.

[37] Wittinghofer A, Herrmann C (1995). Ras-effector interactions, the problem of specificity.. FEBS Lett.

[38] Altin JG, Wetts R, Bradshaw RA (1991). Microinjection of a p21ras antibody into PC12 cells inhibits neurite outgrowth induced by nerve growth factor and basic fibroblast growth factor.. Growth Factors.

[39] Ng NF, Shooter EM (1993). Activation of p21ras by nerve growth factor in embryonic sensory neurons and PC12 cells.. J Biol Chem.

[40] Cowley S, Paterson H, Kemp P, Marshall CJ (1994). Activation of MAP kinase kinase is necessary and sufficient for PC12 differentiation and for transformation of NIH 3T3 cells.. Cell.

[41] Rydel RE, Greene LA (1987). Acidic and basic fibroblast growth factors promote stable neurite outgrowth and neuronal differentiation in cultures of PC12 cells.. J Neurosci.

[42] Taparowsky E, Suard Y, Fasano O, Shimizu K, Goldfarb M (1982). Activation of the T24 bladder carcinoma transforming gene is linked to a single amino acid change.. Nature.

[43] Davies H, Bignell GR, Cox C, Stephens P, Edkins S (2002). Mutations of the BRAF gene in human cancer.. Nature.

[44] Heasley LE, Johnson GL (1992). The beta-PDGF receptor induces neuronal differentiation of PC12 cells.. Mol Biol Cell.

[45] Nguyen TT, Scimeca JC, Filloux C, Peraldi P, Carpentier JL (1993). Co-regulation of the mitogen-activated protein kinase, extracellular signal-regulated kinase 1, and the 90-kDa ribosomal S6 kinase in PC12 cells. Distinct effects of the neurotrophic factor, nerve growth factor, and the mitogenic factor, epidermal growth factor.. J Biol Chem.

[46] Traverse S, Gomez N, Paterson H, Marshall C, Cohen P (1992). Sustained activation of the mitogen-activated protein (MAP) kinase cascade may be required for differentiation of PC12 cells. Comparison of the effects of nerve growth factor and epidermal growth factor.. Biochem J.

[47] Qui MS, Green SH (1992). PC12 cell neuronal differentiation is associated with prolonged p21ras activity and consequent prolonged ERK activity.. Neuron.

[48] Bos JL (1998). All in the family? New insights and questions regarding interconnectivity of Ras, Rap1 and Ral.. Embo J.

[49] von Mering C, Krause R, Snel B, Cornell M, Oliver SG (2002). Comparative assessment of large-scale data sets of protein-protein interactions.. Nature.

[50] Altschuler D, Lapetina EG (1993). Mutational analysis of the cAMP-dependent protein kinase-mediated phosphorylation site of Rap1b.. J Biol Chem.

[51] Hu CD, Kariya K, Okada T, Qi X, Song C (1999). Effect of phosphorylation on activities of Rap1A to interact with Raf-1 and to suppress Ras-dependent Raf-1 activation.. J Biol Chem.

[52] Goldfinger LE (2008). Choose your own path: specificity in Ras GTPase signaling.. Mol Biosyst.

[53] Martin-McCaffrey L, Willard FS, Oliveira-dos-Santos AJ, Natale DR, Snow BE (2004). RGS14 is a mitotic spindle protein essential from the first division of the mammalian zygote.. Dev Cell.

[54] Shu FJ, Ramineni S, Amyot W, Hepler JR (2007). Selective interactions between Gi alpha1 and Gi alpha3 and the GoLoco/GPR domain of RGS14 influence its dynamic subcellular localization.. Cell Signal.

[55] Cho H, Kehrl JH (2007). Localization of Gi alpha proteins in the centrosomes and at the midbody: implication for their role in cell division.. J Cell Biol.

[56] Wohlgemuth S, Kiel C, Kramer A, Serrano L, Wittinghofer F (2005). Recognizing and defining true Ras binding domains I: biochemical analysis.. J Mol Biol.

[57] Howe CL, Mobley WC (2004). Signaling endosome hypothesis: A cellular mechanism for long distance communication.. J Neurobiol.

