# Supplementary figures and images for: Regulator of G-Protein Signaling 14 (RGS14) Is a Selective H-Ras Effector
Source: PLoS One. 2009 Mar 25;4(3):e4884. doi: 10.1371/journal.pone.0004884 (PMC2655719; doi:10.1371/journal.pone.0004884)

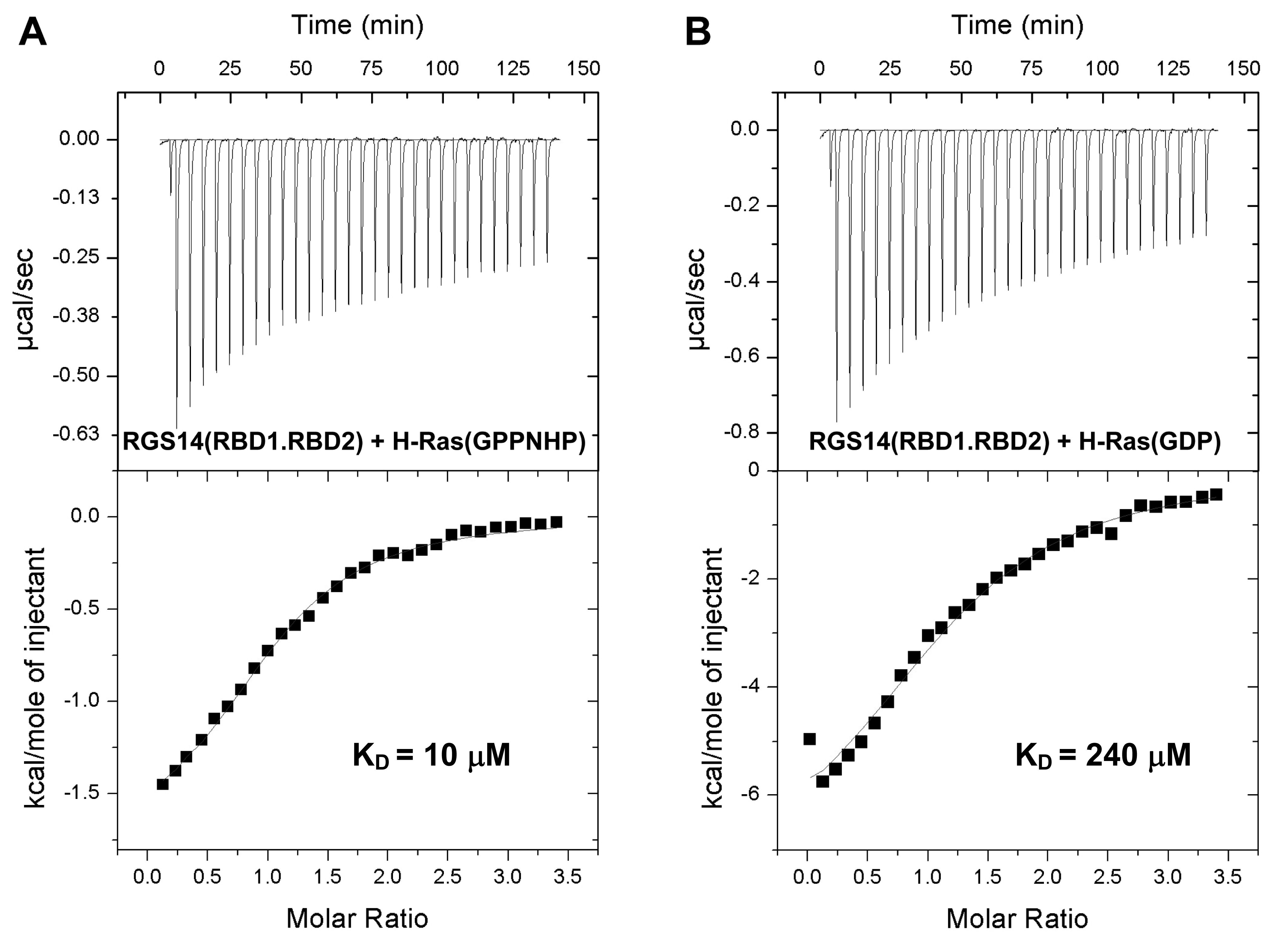

Supplement: Figure S1 — Guanine nucleotide-state selective interaction between H-Ras and RGS14. Isothermal titration calorimetry was used to measure the interaction between H Ras and the isolated Ras-binding domains of RGS14 (“RGS14(RBD1.RBD2)”). A stepwise titration of 300 µM RGS14(RBD1.RBD2) protein into a cell containing 20 µM H Ras(GPPNHP) (A) or H-Ras(GDP) (B) was performed and the heat change accompanying RGS14 injection was detected by comparison with a reference cell. RGS14(RBD1.RBD2) injected into buffer alone was used as a negative control. Heat changes were plotted against the molar ratio of H Ras to RGS14(RBD1.RBD2) protein and analyzed using non-linear regression (see Table 1 of the main manuscript for data analysis parameters). Data was fit by applying a one-site binding model involving exothermic reaction phases (negative enthalpy changes) with favorable free energy changes. Analysis of the data indicates that complete saturation of the binding site is not achieved. This is likely due to the high dissociation rate of the complex. (0.66 MB TIF) [file pone.0004884.s001.tif]

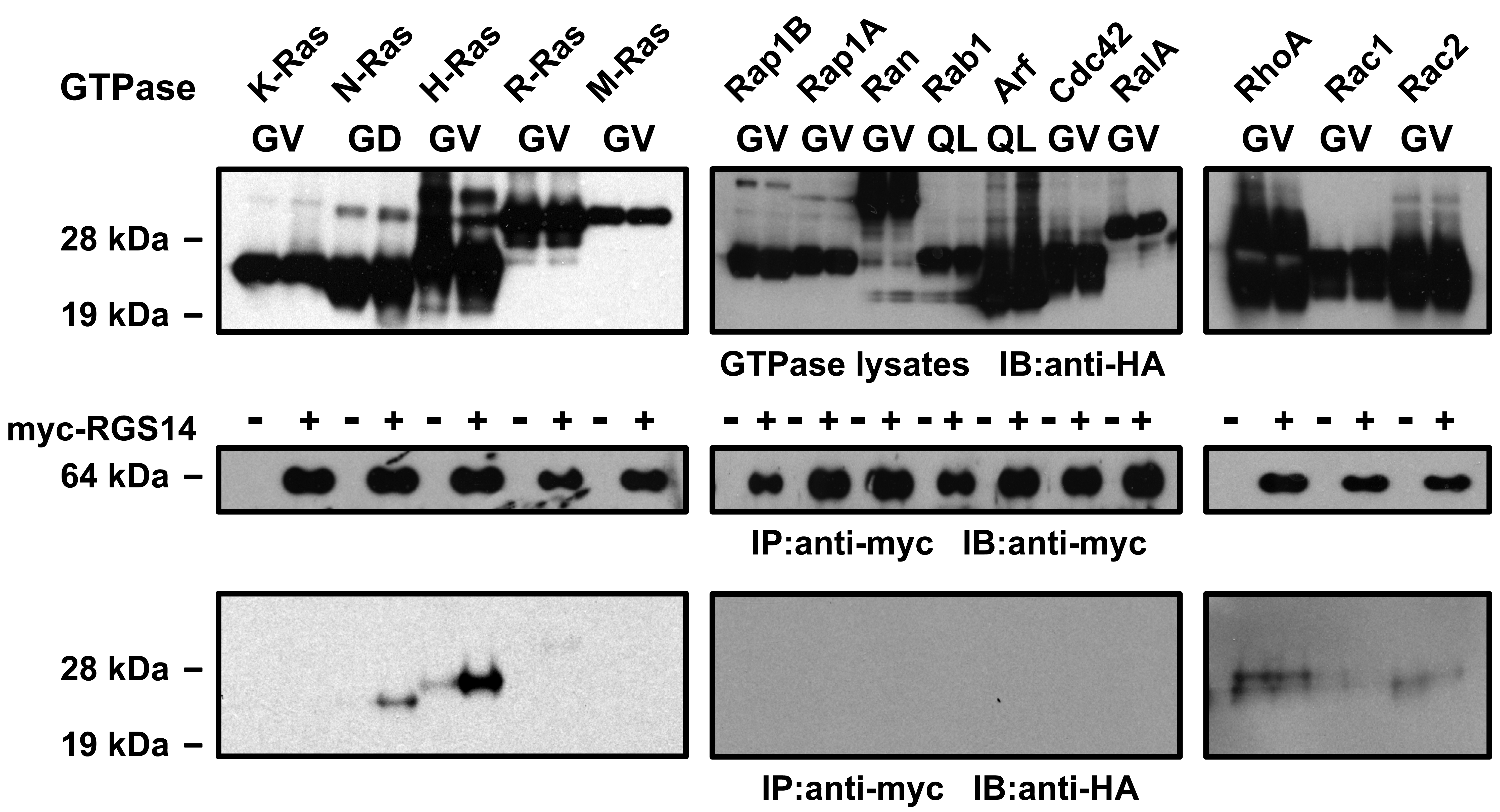

Supplement: Figure S2 — RGS14 selectively interacts with H-Ras and not other small GTPases in cells. HEK293T cells were transfected with plasmids encoding full-length myc-RGS14 and various mutationally-activated (“GV”, “GD”, or “QL”), HA-epitope tagged GTPases. Cell lysates were immunoprecipitated (IP) with anti-myc antibodies. Total lysates and precipitates were immunoblotted (IB) with indicated antibodies. “Arf GTPase” denotes the use of Arf1. (3.18 MB TIF) [file pone.0004884.s002.tif]

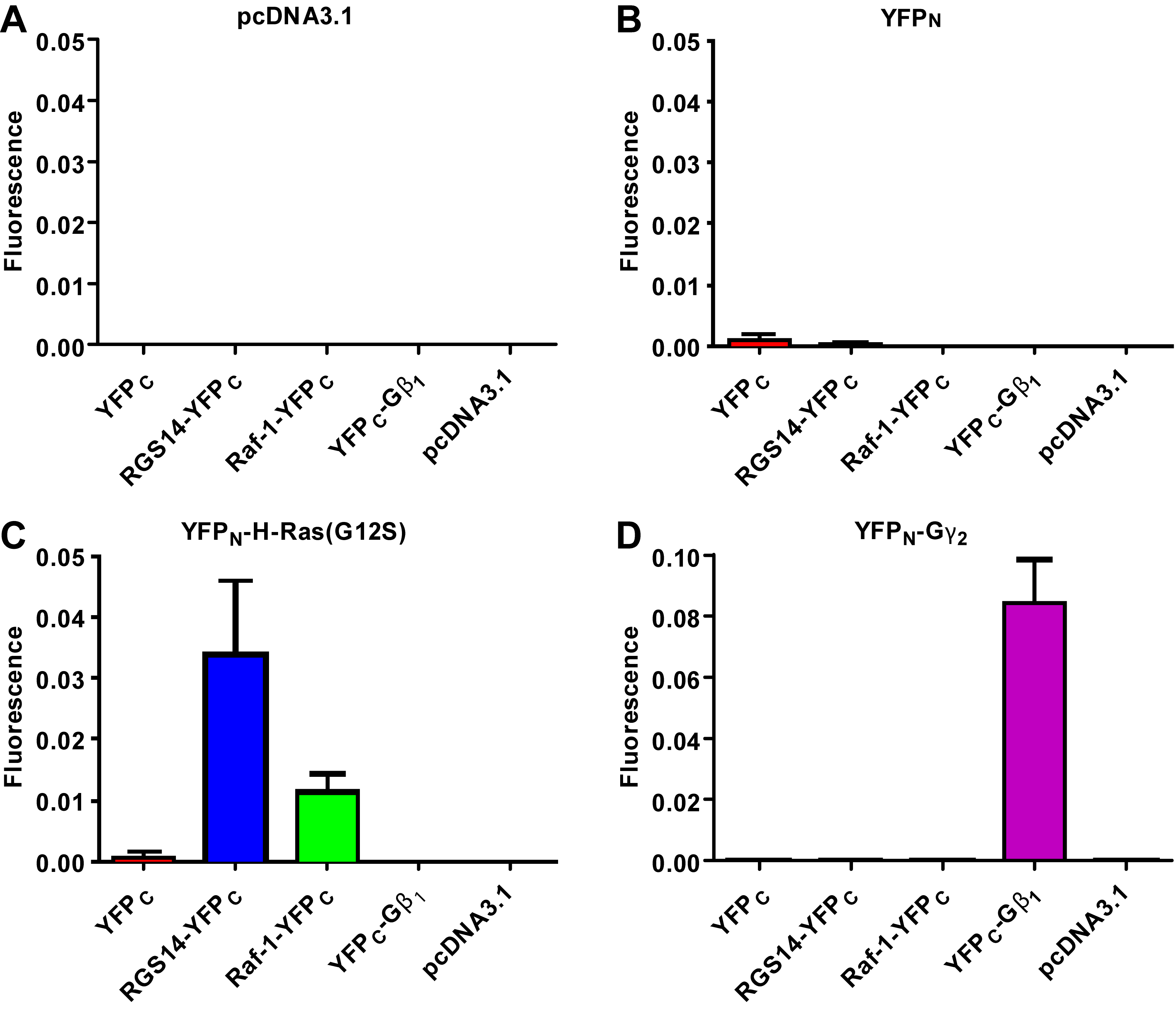

Supplement: Figure S3 — Specificity of fluorescence complementation between H-Ras(G12S) and RGS14. HEK293T cells were co-transfected with cDNAs encoding the empty vector pcDNA3.1, the N-terminal (amino acids 1–159) and C-terminal (amino acids 159-239) fragments of Yellow Fluorescent Protein (YFPN and YFPC), and indicated proteins fused to YFPN and YFPC. 48 hours after transfection, cells were analyzed by epifluorescence microscopy, and fluorescence was quantified using image analysis as described in the Experimental section. (A) Transfection of the YFPC vector or YFPC-fusion constructs does not result in measurable fluorescence in the absence of YFPN co-transfection. (B) YFPN alone does not complement YFPC nor YFPC-fusion constructs. (C) YFPN-H-Ras(G12S) complements both RGS14-YFPC and Raf-1-YFPC but not YFPC nor YFPC-Gβ1. (D) YFPN-Gγ2 complements YFPC-Gβ1 but not YFPC, RGS14-YFPC, nor Raf-1-YFPC. (0.86 MB TIF) [file pone.0004884.s003.tif]

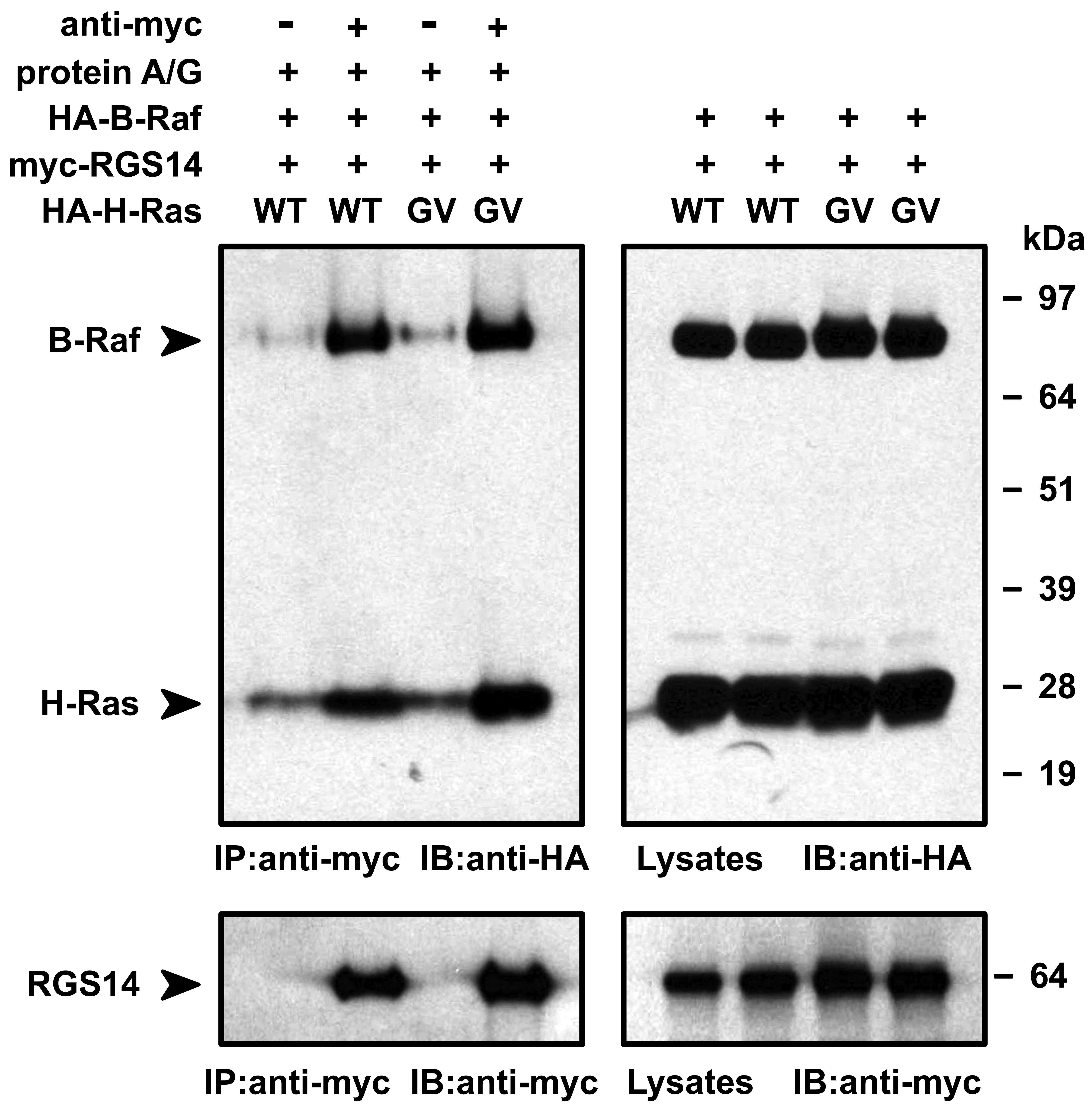

Supplement: Figure S4 — Specificity of H-Ras/RGS14/B-Raf complex formation. HEK293T cells were transfected with plasmids encoding full-length myc-RGS14, HA-B-Raf, and either wild-type or G12V HA-H-Ras. Cell lysates were immunoprecipitated (IP) with anti-myc antibodies and protein A/G agarose, as indicated. Total lysates and immunoprecipitates were immunoblotted (IB) with indicated antibodies. (3.08 MB TIF) [file pone.0004884.s004.tif]

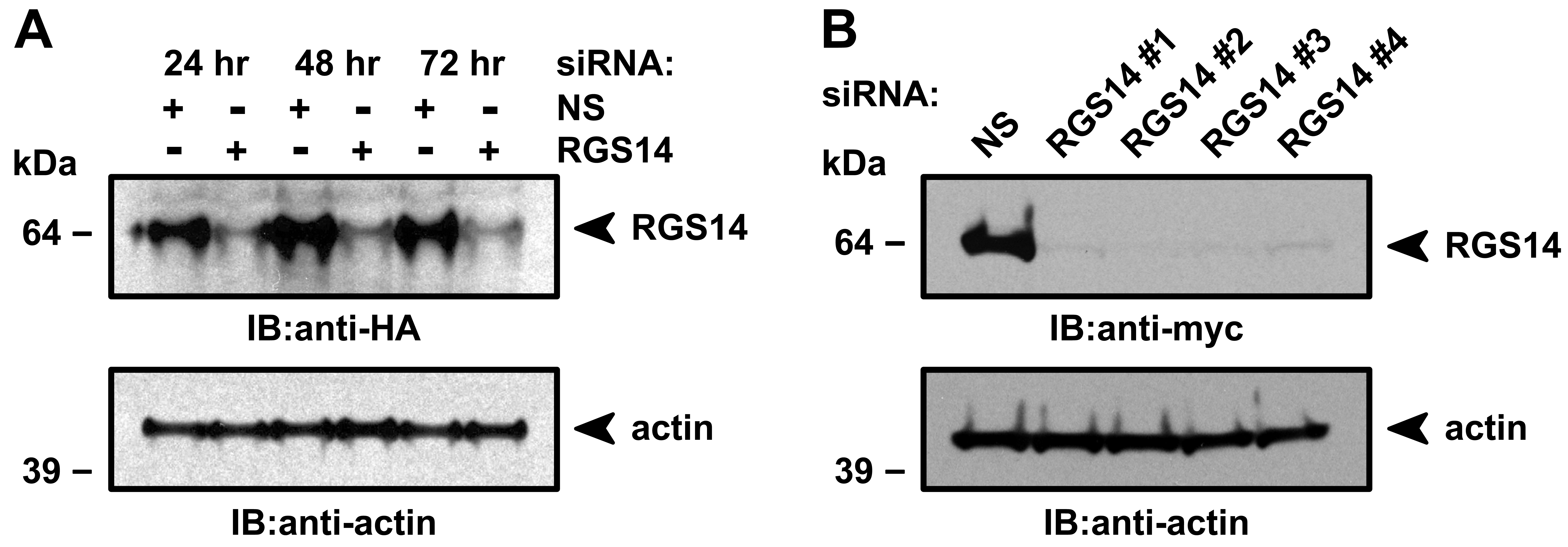

Supplement: Figure S5 — Specificity and efficacy of rat RGS14 siRNAs (I). (A) HEK293T cells were transfected with HA-epitope tagged RGS14 expression vector and then 6 hours later transfected with control non-specific (NS) siRNA or a pool of four RGS14 siRNAs. 24, 48, and 72 hours later, RGS14 expression level was analyzed by immunoblot (IB) with anti-HA. Samples were immunoblotted with anti-actin antibodies as a control for total protein levels. (B) HEK293T cells were transfected with myc-epitope tagged RGS14 expression vector and then 6 hours later transfected with control non-specific (NS) siRNA or four independent RGS14 siRNA duplexes (#1-4) that constitute the siRNA SMARTpool used in panel A. 48 hours later RGS14 expression level was analyzed by immunoblot with anti-myc antibodies. Samples were immunoblotted with anti-actin antibodies as a control for total protein levels. (1.89 MB TIF) [file pone.0004884.s005.tif]

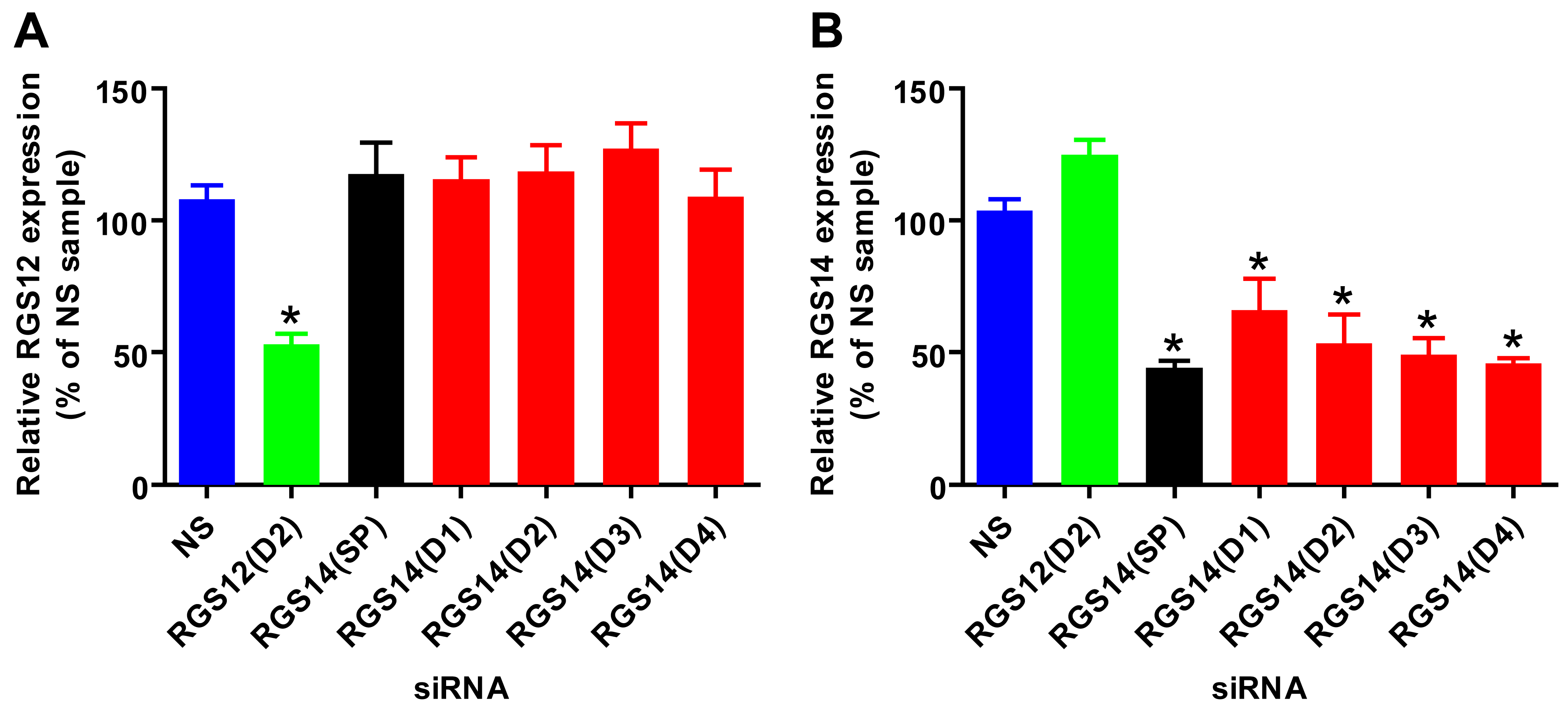

Supplement: Figure S6 — Specificity and efficacy of rat RGS12 and RGS14 siRNAs (II). PC-12 cells were transfected with control non-specific (NS) siRNA, RGS12(D2) siRNA (duplex 2 from ref. 20), a SMARTpool (SP) of four RGS14 siRNA duplexes, or the four individual constituent siRNA duplexes (D1, D2, D3, and D4) which comprise the SMARTpool. Forty eight hours later, cells were harvested, RNA was extracted, and RGS12 (A) and RGS14 (B) expression levels were measured by quantitative real-time PCR (as performed by the Gene Expression Core of the UNC Dept. of Laboratory Medicine and Pathology, directed by Dr. Hyung-Suk Kim). RGS12 and RGS14 data were normalized for relative expression levels using the 2-(Ct) method with β-actin as the internal control. Data is presented as relative expression compared to non-specific (NS) siRNA treated samples. Statistical significance was determined using ANOVA with Dunnett's multiple comparison test (* denotes P<0.5 vs NS siRNA samples). (0.30 MB TIF) [file pone.0004884.s006.tif]

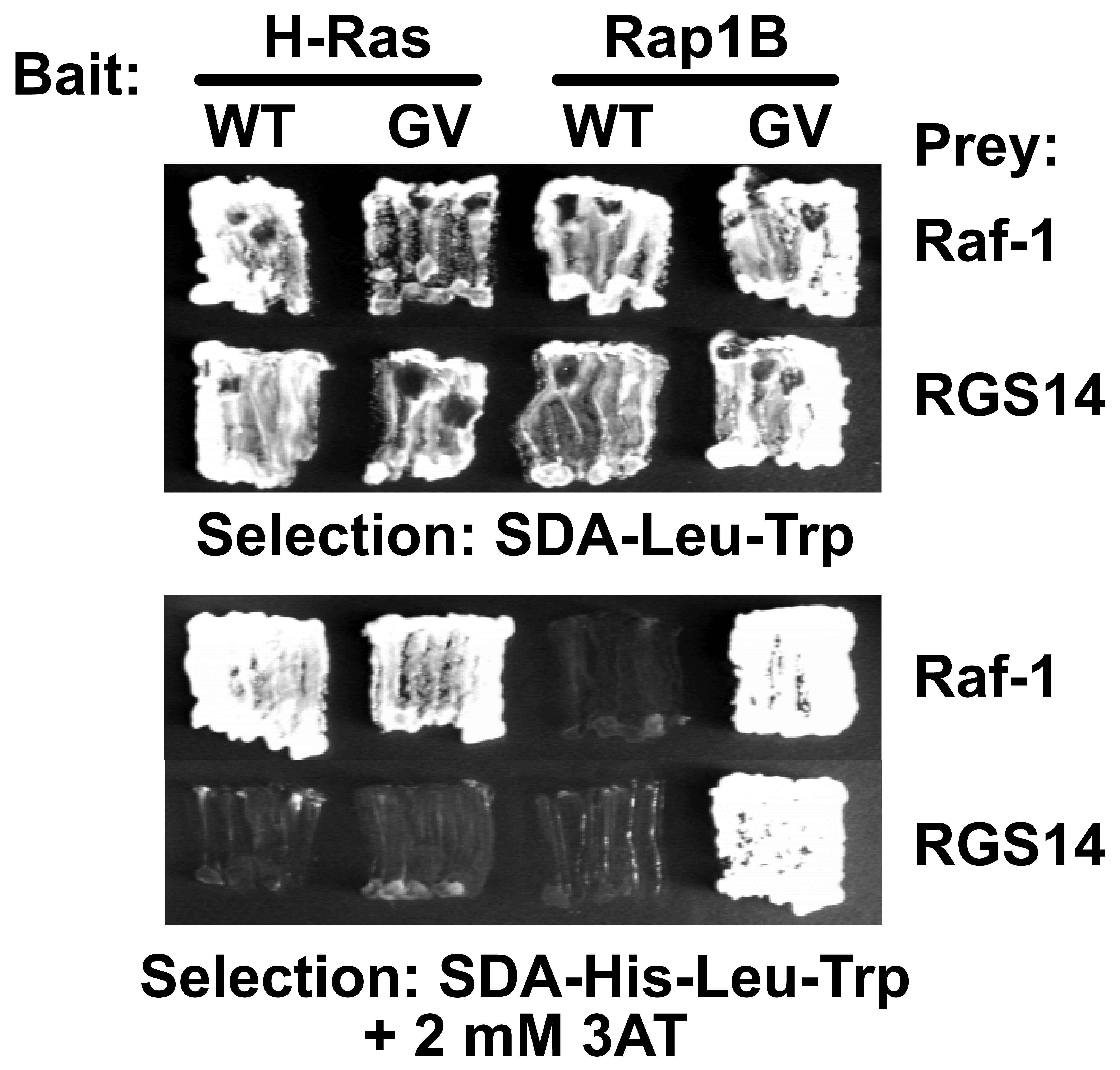

Supplement: Figure S7 — Yeast two-hybrid analysis of interactions between RGS14 and Ras-family GTPases. Yeast were co-transformed with bait plasmids encoding indicated GTPase fusions with the Gal4p DNA binding domain and prey plasmids encoding either Raf-1 or RGS14 fused to the Gal4p activation domain. Wild-type (WT) or glycine-12-to-valine (GV) mutationally-activated GTPases were used to test for activation-dependent binding to the Ras-binding domain (RBD) of Raf-1 (amino acids 50–131) and the tandem RBDs and GoLoco motif of RGS14 (amino acids 263–544). Yeast were plated on synthetic defined agar (SDA), lacking leucine (-Leu, to select for the pACT-II plasmid containing the LEU2 gene), and tryptophan (-Trp, to select for the pGBT9 plasmid containing the TRP1 gene). Growth on SDA-Leu-Trp demonstrates incorporation of bait and prey plasmids (top panel). Growth on SDA-Leu-Trp-His in the presence of the histidine biosynthesis inhibitor 3-amino-1,2,4-triazole (3AT) indicates a positive protein-protein interaction. (2.04 MB TIF) [file pone.0004884.s007.tif]
