# Supplementary material for: Regulator of G-Protein Signaling 14 (RGS14) Is a Selective H-Ras Effector
Source: PLoS One. 2009 Mar 25;4(3):e4884. doi: 10.1371/journal.pone.0004884 (PMC2655719; doi:10.1371/journal.pone.0004884)
Supplement: Table S1 — DNA constructs created and obtained for use in this study. (0.10 MB PDF) [file pone.0004884.s008.pdf]

DNA constructs created and obtained for use in this study.

Plasmid	Source or methods used for construction
pGEX4T2 and TEV protease site derivative pGEX4TEV2	GE Healthcare and [1]
pACTII	Clontech (Mountain View, CA)
pcDNA3.1LIC(HA), pcDNA3.1LIC(myc), pET28LIC(GST-TEV), pET28LIC(His_6-TEV)	Jason Snyder, Svetlana Gershberg, and Janeen Vanhooke (UNC-CH, Pharmacology)
pcDNA3.1, rat RGS14, N-terminal 2xHA tag	[2]
Human RGS14, *I.M.A.G.E.* clone 4547415	ATCC (Manassas, VA)
Mouse RGS14 (GenBank BC030321) and mouse RGS12 (GenBank BC040396)	Open Biosystems (Huntsville, AL)
pGEX4TEV2(rat RGS14-His_6) prokaryotic expression vector	[3]
pGEXTEV2 prokaryotic expression vector encoding mouse RGS14 RBD1(aa 302-378), RBD2(aa 366-450), and RBD1.RBD2(aa 302-450)	Constructed using heterostagger PCR, as described in [4]
pNIC-SGC(RGS14(RBD1.RBD2)) prokaryotic expression vector; encodes amino acids 300-446 of RGS14 with an N-terminal His_6 tag and TEV protease cleavage site	Constructed from *I.M.A.G.E.* clone 4547415, using PCR and LIC [5]
pcDNA3.1-based vector encoding full length rat RGS14 with an N-terminal myc-His_6 epitope tag (MAARGHPFEQKLISEEDLNMHTGHHHHHHCVNS)	Constructed using heterostagger PCR and *EcoRI/NotI* restriction sites as described in [1]
pcDNA3.1LIC(HA) full-length mouse RGS12 and mouse RGS14	Constructed using PCR and LIC, as described [6,7]
pACTII yeast expression vector encoding Gal4p activation domain fusion proteins of human c-Raf-1(aa 50-131) or rat RGS14(aa263-544)	Constructed by PCR to generate amplicons with 5’-*EcoRI* and 3’-*SalI* restriction sites. Amplicons were digested with *EcoRI*+*SalI* and ligated into *EcoRI*+*XhoI* digested pACTII.
pGEX4T2 prokaryotic expression vector encoding human c-Raf-1(aa 50-131)	Constructed by PCR to generate an amplicon with 5’-*EcoRI* and 3’-*SalI* restriction sites. Amplicons were digested with *EcoRI*+*SalI* and ligated into *EcoRI*+*XhoI* digested pGEX4T2.
pcDNA3.1, HA-tagged human B-Raf and human c-Raf-1-FLAG	K.L. Guan (University of Michigan, Ann Arbor, MI)
pUSEamp, HA-tagged rat MEK1	Upstate/Millipore (Billerica, MA)
pcDNA3, HA-tagged ERK1	Channing Der (UNC-CH, Pharmacology)
Human A-Raf with an N-terminal FLAG tag in pCMV-FLAG-6b	Jeffrey Frost (UT Health Science Center, Houston, TX) [8]
Human B-Raf with a C-terminal FLAG tag in pLNCX	Deborah Morrison (NCI, Frederick, MD)
pCGN HA-tagged H-Ras G12V, K-Ras G12V, and N-Ras G12D	Channing Der (UNC-CH, Pharmacology)

pBABE, H-Ras(G12V) and B-Raf(V600E)	Channing Der (UNC-CH, Pharmacology)
pcDNA3.1 expression constructs for untagged GTPases (RalA, Arf1, Rab1A, Rap2A)	UMR cDNA resource (www.cdna.org)
pcDNA3.1(Rap2A G12V) untagged	This study; created by mutagenesis of the wild-type UMR clone.
pcDNA3.1 expression constructs for 2xHA tagged GTPases (RalA, Arf, and Rab1A)	This study; generated via restriction of wild-type ORFs with *BamHI/XhoI*, ligation of resultant fragment into a pcDNA3.1 variant encoding N-terminal 2xHA epitope tag [9]
pcDNA3.1 expression constructs for 2xHA tagged GTPases (RalA G23V, Arf1 Q70L, and Rab1A Q76L)	This study; generated via site directed mutagenesis of wild-type clones.
pcDNA3.1 expression constructs for 3xHA tagged GTPases (Rap1A G12V, Rap1B G12V, H-Ras WT, H-Ras G12V, M-Ras GV, R-Ras GV, RanGV, Cdc42 GV, RhoA GV, Rac1 GV, Rac2GV)	UMR cDNA resource (www.cdna.org)
FLAG-tagged Rap2B wild-type and G12V in pCMV2	Lawrence Quilliam (IUPUI, Indianapolis, IN)
pPROEXHTb(human H-Ras) prokaryotic expression vector	Jason Snyder (UNC-CH, Pharmacology)
pGBT9 (wild-type and GV, H-Ras)	Michael Wigler and Linda Van Aelst (Cold Spring Harbor Laboratory, NY) [10]
pGBT9 (wild-type and GV, Rap1B)	Gil White (Blood Research Institute, Milwaukee, WI) [11]
Mammalian expression construct encoding Venus EYFP(C-terminal fragment, amino acids 159-239) fused to Raf-1 and Venus EYFP(N-terminal fragment, amino acids 1-159) fused to H-Ras(G12S)	John Westwick (Odyssey Thera) [12]
pcDNA3.1LIC(HA) encoding an N-terminal HA tag, a linker sequence SGILYFQSNAP, RGS14(rat; amino acids 2-544), the linker sequence RIGSGSR, and then Venus EYFP(C-terminal fragment, amino acids 159-239)	Created for this study using PCR and LIC [6,7]
pcDNA3.1 expression vectors encoding the N-terminal fragment (amino acids 1-159) of Venus YFP fused to $G\gamma_2$ and the C-terminal fragment (amino acids 159-239) of Venus YFP fused to $G\beta_1$.	Nevin Lambert (Medical College of Georgia, Augusta, GA)

References for Supplementary Table 1

1. Kimple RJ, Willard FS, Hains MD, Jones MB, Nweke GK, et al. (2004) Guanine nucleotide dissociation inhibitor activity of the triple GoLoco motif protein G18: alanine-to-aspartate mutation restores function to an inactive second GoLoco motif. Biochem J 378: 801-808.

2. Martin-McCaffrey L, Willard FS, Oliveira-dos-Santos AJ, Natale DR, Snow BE, et al. (2004) RGS14 is a mitotic spindle protein essential from the first division of the mammalian zygote. Dev Cell 7: 763-769.

3. Martin-McCaffrey L, Willard FS, Pajak A, Dagnino L, Siderovski DP, et al. (2005) RGS14 is a microtubule-associated protein. Cell Cycle 4: 953-960.

4. Willard FS, Kimple AJ, Johnston CA, Siderovski DP (2005) A direct fluorescence-based assay for RGS domain GTPase accelerating activity. Anal Biochem 340: 341-351.

5. Graslund S, Nordlund P, Weigelt J, Hallberg BM, Bray J, et al. (2008) Protein production and purification. Nat Methods 5: 135-146.

6. Willard FS, Low AB, McCudden CR, Siderovski DP (2007) Differential G-alpha interaction capacities of the GoLoco motifs in Rap GTPase activating proteins. Cell Signal 19: 428-438.

7. Stols L, Gu M, Dieckman L, Raffen R, Collart FR, et al. (2002) A new vector for high-throughput, ligation-independent cloning encoding a tobacco etch virus protease cleavage site. Protein Expr Purif 25: 8-15.

8. Tran NH, Wu X, Frost JA (2005) B-Raf and Raf-1 are regulated by distinct autoregulatory mechanisms. J Biol Chem 280: 16244-16253.

9. Snow BE, Krumins AM, Brothers GM, Lee SF, Wall MA, et al. (1998) A G protein gamma subunit-like domain shared between RGS11 and other RGS proteins specifies binding to Gbeta5 subunits. Proc Natl Acad Sci U S A 95: 13307-13312.

10. Van Aelst L, Barr M, Marcus S, Polverino A, Wigler M (1993) Complex formation between RAS and RAF and other protein kinases. Proc Natl Acad Sci U S A 90: 6213-6217.

11. Peterson SN, Trabalzini L, Brtva TR, Fischer T, Altschuler DL, et al. (1996) Identification of a novel RalGDS-related protein as a candidate effector for Ras and Rap1. J Biol Chem 271: 29903-29908.

12. MacDonald ML, Lamerdin J, Owens S, Keon BH, Bilter GK, et al. (2006) Identifying off-target effects and hidden phenotypes of drugs in human cells. Nat Chem Biol 2: 329-337.
